# Effect of long-term temperature stress on the intestinal microbiome of an invasive snail

**DOI:** 10.3389/fmicb.2022.961502

**Published:** 2022-08-29

**Authors:** Shuxian Li, Zijin Qian, Shuo Gao, Wenjia Shen, Xuexia Li, Hong Li, Lian Chen

**Affiliations:** ^1^Co-Innovation Center for Sustainable Forestry in Southern China, College of Biology and the Environment, Nanjing Forestry University, Nanjing, China; ^2^Jiangsu Key Laboratory for Biodiversity and Biotechnology, College of Life Sciences, Nanjing Normal University, Nanjing, China

**Keywords:** *Pomacea canaliculata*, temperature, 16S rRNA, microbiome, invasive snail

## Abstract

The gut microbiome is vital to the physiological and biochemical functions of the host, and changes in the composition of these microbial communities may affect growth and adaptability to the environment. *Pomacea canaliculata* is an invasive freshwater snail which has become a serious agricultural pest. Temperature adaptation is considered an important reason for the widespread distribution of this species. To date, the contribution of the gut microbes to host fitness of *P. canaliculata* during long-term temperature stress is not well understood. In this study, the morphological changes and intestinal microbiome of *P. canaliculata* under long-term stress at low temperature (15°C) and high temperature (35°C) were investigated with laboratory experiments. Compared with control group (25°C), the alpha diversity increased and pathogenic bacteria enriched changed under high and low temperature stress. The effect of high temperature stress on the intestinal microbiome of *P. canaliculata* was more significant than that of low temperature stress. A sustained high temperature environment led to an increase in the abundance of pathogenic bacteria, such as *Aeromonas* and *Enterobacter*, and a decrease in the abundance of immune-related bacteria such as Bacteroidetes, Firmicutes, and *Lactococcus*. These intestine microbiome changes can increase the risk of diseases like intestinal inflammation, and lead to more deaths at high temperature environments. In addition, with the extension of stress time from 14 to 28 days, the beneficial bacteria such as Bacteroidetes, Firmicutes, and *Lactococcus* were significantly enriched, while potential pathogenic bacteria such as *Pseudomonas*, *Acinetobacter*, *Shivalella*, and *Flavobacterium* decreased, suggesting that intestinal microbiota may play an important role in host response to heat stress. These results are consistent with previously reported results that the survival rate of both male and female *P. canaliculata* no longer significantly reduced after 21 days of high temperature stress, suggesting that the surviving *P. canaliculata* had gradually adapted to high temperature environments under long-term high temperature stress.

## Introduction

*Pomacea canaliculata* (Caenogastropoda: Ampullariidae) is an invasive freshwater snail originating from South America that has been introduced outside its native range on occasion to other locations (Hayes et al., 2008), including Asia, Europe, and both North and South America, and it has become a serious agricultural pest that can damage crops and wetland ecosystems (Cowie, 2002; Hayes et al., 2015). During the invasion process of *P. canaliculata*, a variety of ecological adaptation mechanisms have occurred to cope with various environmental stresses, among which temperature adaptation is an important cause of the widespread distribution of this species (Yusa et al., 2006). Previous studies have shown that tolerance to low and high temperature environments is a key factor for the adaptation of *P. canaliculata* to harsh environments and successful expansion of its range of activities (Zheng et al., 2012; Yoshida et al., 2014).

Temperature is an important abiotic factor that affects the physiological state of animals, especially for aquatic organisms (Sepulveda and Moeller, 2020). It is an important environmental variable that affects both the physiological and biochemical reactions of aquatic organisms, thereby controlling their metabolic reaction rate and affecting their growth and development (Kelley, 2014; Jiang et al., 2016; Sepulveda and Moeller, 2020). To date, it is widely recognized that several aspects of the life cycle of *P. canaliculata*, including growth, feeding, crawling, aerial respiration, reproduction and survival, can be affected by temperature (Matsukura and Wada, 2007; Seuffert et al., 2010). *P. canaliculata* died at low temperatures close to 0°C (Matsukura et al., 2009). During winter most *P. canaliculata* are inactive but not in a deep lethargic state, and at 13–15°C, half are active. Laboratory test showed that the active and feeding time of *P. canaliculata* increased significantly as the temperature increased from 10 to 30°C (Seuffert et al., 2010), and their activity level and feeding ability peaked between 25 and 32°C (Heiler et al., 2008; Seuffert et al., 2010). Although *P. canaliculata* had no mortality at 15 and 20°C, their growth rate was low, whereas at 25, 30, and 35°C the snails grew faster but displayed a reduction in survival (Seuffert and Martín, 2013). At temperatures above 35°C, however, the growth rate of *P. canaliculata* decreased significantly (Seuffert and Martín, 2013). The optimal temperature for the hatching of *P. canaliculata* eggs is about 30°C, with no eggs are laid above 35°C, and no reproductive activities are carried out below 15°C (Seuffert and Martín, 2017). The frequency and duration of side crawling of *P. canaliculata* significantly decreased when the water temperature was decreased from 25 to 20°C or 15°C, while the ventilation behavior and food consumption increased when the water temperature was increased from 25 to 30°C (Bae et al., 2021).

The gut microbiome, also known as the “animal second genome,” has co-evolved with animals, interacts with each other, and is capable of significantly affecting the physiology, development and health of its hosts (McFall-Ngai et al., 2013). The community structure and diversity of the gut microbiome are affected by the host’s own physiological conditions. The gut microbiome can also participate in the digestion and absorption of host nutrients as well as energy metabolism, and can function to regulate the expression of host genes, material metabolism, and tissue and organ development (Flint et al., 2008; Hall et al., 2017). The gut microbiota is in a dynamic balance, and various host and environmental factors can affect its diversity and community structure (Zhao et al., 2021), such as host gender (Ma et al., 2018), health status (Chandler et al., 2014), diet (Cardoso et al., 2012), season (Pierce et al., 2016), and temperature (Li et al., 2019; Sepulveda and Moeller, 2020).

Temperature is considered one of the main factors affecting the gut microbial community (Sepulveda and Moeller, 2020) and long-term seasonal changes in temperature may lead to adaptive changes in the composition of the gut microbiome. The gut and pallial fluid microbiome of the American oyster (*Crassostrea virginica*) are affected significantly by water temperature, which might be attributed the decreased water temperatures in winter that leads to decreased metabolic activity in many bacteria and oysters due to reduced physical activity (Pierce et al., 2016). Gut microbial communities may also be affected by temperature on shorter timescales, such as extreme weather events (heat waves, cold snaps, etc.) (Gao et al., 2012). Studies showed that the composition of the gut microbial community of the red-backed salamander (*Plethodon cinereus*) differed under the three temperatures (10, 15, and 20°C). High temperature stress led to a decrease in the relative abundance of disease-resistant bacteria and an increase in the relative abundance of pathogenic bacteria (Fontaine et al., 2018). If the gut microbiota of some species is more plastic than others, they may exhibit greater phenotypic plasticity in new environments, and such a feature is often found in invasive organisms (Davidson et al., 2011). Studies have shown that ambient temperature can alter gut microbial diversity and composition in ectothermic animals (Bestion et al., 2017; Huyben et al., 2018; Fontaine and Kohl, 2020).

In this study, we investigated the effects of high temperature and low temperature stress on the growth and development of *P. canaliculata* along with the response of the intestinal microbiome to the long-term temperature stress. We hope that revealing the ability of invasive snails to quickly adapt to environmental changes and the mechanism of successful invasion will provide new ideas and a scientific basis for the prevention and effective management of invasive organisms.

## Materials and methods

### Sample collection

The eggs of *P. canaliculata* were collected from Taihu Wetland Park located in Kunshan (N 30.916°, E 120.403°), Jiangsu, China, in June, 2021. These eggs were brought back to the lab and hatched using a climate chamber (25°C with a cycle of 14 h-light: 10 h-dark; the humidity was 80%).

Species were identified based on the morphological characteristics of the egg mass combined with molecular experiments. Two to three eggs were randomly selected from each egg mass to extract genomic DNA, amplify the mitochondrial cytochrome c oxidase subunit I (COI) gene fragment (Yang et al., 2018), and sequence the target PCR product. After the eggs were confirmed to be *P. canaliculata*, the eggs masses were placed in an incubator to hatch. The hatchlings used in the experiment were put into plastic boxes (36 × 25 × 12 cm) containing aerated water and kept at 25°C. During the course of the experiment, *P. canaliculata* were fed with lettuce leaves every day, the aerated water was changed every 2 days.

### Temperature treatment and morphometric measurement

*Pomacea canaliculata* of the same age (three and half a month) with similar length (female: 26.47 ± 0.96 mm; male: 25.96 ± 1.16 mm) were selected for temperature stress experiments using different temperatures: high temperature stress (35°C), low temperature stress (15°C) and control (25°C). Female and male *P. canaliculata* were divided to create a total of six experimental groups including high temperature group (HF), low temperature group (LF), and control group (CF) for females, and high temperature group (HM), low temperature group (LM), and control group (CM) for males. Three parallel repetitions were performed in each group, with 20 snails were placed in each repetition. The temperature stress experiment was conducted in an artificial climate box with a 14 h-light: 10 h-dark photoperiod and an 80% humidity. During the experiment, aerated water was used and changed every 2 days, with replacement water placed into a corresponding climate box 1 day in advance to make temperature consistent until the end of the experiment. Fresh lettuce leaves were fed every day.

All *P. canaliculata* were marked with a marking pen for morphological data measurement of the snails during the experiment. From the beginning of the experiment (0 days) to the end of the experiment (28 days), the behavior and activities of *P. canaliculata* were observed at a fixed time three times a day. The number of dead snails was recorded and these dead individuals were removed in a timely manner. Acupuncture and rehydration methods were used to identify the survival, suspended animation, and death (Bernatis et al., 2016). Morphological data measurements were performed every 7 days. The wet weight of the snails was measured and recorded using an electronic balance (Mettler Toledo, 0.001 g). Vernier calipers were used (Mitutoyo, 0.01 mm) to measure and record the length and width of the snail shells. The relevant formulas are as follows:

(1) Survival rate (%) = (Number of surviving snails/total number of snails) × 100% (Seuffert and Martín, 2013)
(2) Specific growth rate (%) = [(lnW2-lnW1)/(t2-t1)] × 100% (Ruppert et al., 2016)
(3) Weight gain rate (%) = (W2-W1)/W1 × 100% (Júnior et al., 2013)
(4) Growth rate of snail length (%) = (SL2-SL1)/SL1 × 100% (Bernatis et al., 2016)

In the above formula, W1 and W2 are the wet weights of *P. canaliculata* at time t1 and t2, SL1 and SL2 are the shell lengths of *P. canaliculata* at time t1 and t2, respectively.

### Sampling

On the 14th day (14 days) and 28th day (28 days) after the start of the temperature stress experiment, five snails were randomly selected from the parallel replicates of the six temperature groups, for a total of 60 snails. The coiled gut content was extracted from the *P. canaliculata* according to Chen L. et al. (2021). The foot muscle of each of the sampling snails was isolated to further identify the *P. canaliculata* by amplifying COI gene using LCO1490/HCO2198 as described by Hayes et al. (2009). PCR amplification products were sequenced by Shanghai Sangon Biological Engineering Technology & Services Co., Ltd (Shanghai, China).

The group information of the 60 snails was as follows. The samples collected on the 14th day (14 days) included the female snail high temperature group (HF14), female snail low temperature group (LF14), female snail control group (CF14), male snail high temperature group (HM14), male snail low temperature group (LM14), and male snail control group (CM14). The samples collected on the 28th day (28 days) included the female snail high temperature group (HF28), female snail low temperature group (LF28), female snail control group (CF28), male snail high temperature group (HM28), male snail low temperature group (LM28), and male snail control group (CM28). Each group contained five snails.

Total DNA was extracted from the intestinal contents of 60 snails in small tubes using the FastDNA^®^ Kit (MP 149 Biomedicals, Santa Ana, CA, United States). The experiments were carried out using universal primers in the V3-4 region of the 16S rRNA gene. The specific primer sequences were as follows: 341F (5′-CCTAYGGGRBGCASCAG-3′), 806R (5′-GGACTACNNGGGTATCTAAT-3′) (Dao et al., 2021). PCR products were detected using 2% agarose gel. The TruSeq Nano DNA LT Library Prep Kit (Illumina, United States) was used to build the library according to the manufacturer’s protocol. Then the PCR products were quantified using the Agilent High Sensitivity DNA Kit (Beijing, China). After quality assessment and quantification, amplicons were pooled and sequenced according to standard protocols using the Illumina Novaseq platform by Novo-gene Co. Ltd. (Beijing, China).

### Data analysis

Microbiome bioinformatics analysis was performed using QIIME2 (version 2019.4)^1^ (Bolyen et al., 2019). Qiime cutadapt trim-paired was used to excise the primer fragment of the sequence and remove the sequences that did not match the primer. DADA2 was selected based on the qiime dada2 denoise-paired for quality control, denoising, splicing, and removing chimeras in the sequence (Callahan et al., 2016). After denoising all the libraries, the amplicon sequence variants (ASVs) signature sequences and ASV tables were merged, and singleton ASVs were removed. The sequences were aligned and species were annotated against the SILVA SSU rRNA database (version 132)^2^ using RDP Native Bayesian Classifier (version 2.11) (Quast et al., 2013; Bokulich et al., 2018).

Sequence data analysis was mainly performed using QIIME2 and R package (version 3.2.0). The ASV table of QIIME2 was used to calculate the alpha diversity index of the ASV level, including the Shannon-wiener diversity index, Simpson diversity index, Chao1 richness estimator, observed species, and Goods_coverage index, the data are expressed in the form of a box plot. The alpha diversity indices were compared using the Kruskal–Wallis H, and *P* < 0.05 indicates a significant difference.

Non-metric multidimensional scaling (NMDS) and unweighted pair-group method with arithmetic means (UPGMA) were used for beta diversity analysis. Analysis of similarity (ANOSIM) (Clarke, 1993; Warton et al., 2012) was used to assess the significance of microbial community structure differentiation between groups. Mantel tests based on spearman’s correlation analysis were used to determine the correlations between temperature, stress time and microbiota variation, respectively. NMDS ordinations was analyzed based on the Bray-Curtis dissimilarity matrices in the “vegan” package in R (Dixon, 2003).

Morphological data were analyzed with the SPSS 25.0 statistical package, and Kolmogorov–Smirnov and Bartlett tests were used to test data normality and homogeneity respectively. Two-way repeated-measures ANOVA was used to analyze the effects of temperature and sex on the growth rate of snail length, weight gain rate and specific growth rate of *P. canaliculata*, with temperature treatments and sex as the fixed factors, and time as the within-subject factor. The relative abundances of important dominant bacterial phyla in the high temperature group, low temperature group and control group were compared based on one-way ANOVA. The differences between treatments in multiple samples were tested using Duncan’s multiple comparisons. All descriptive statistical values are expressed as mean ± standard error (SE), and the significance level was set at α = 0.05.

## Results

### Effect of temperature on the growth and development of *Pomacea canaliculata*

**FIGURE 1:**
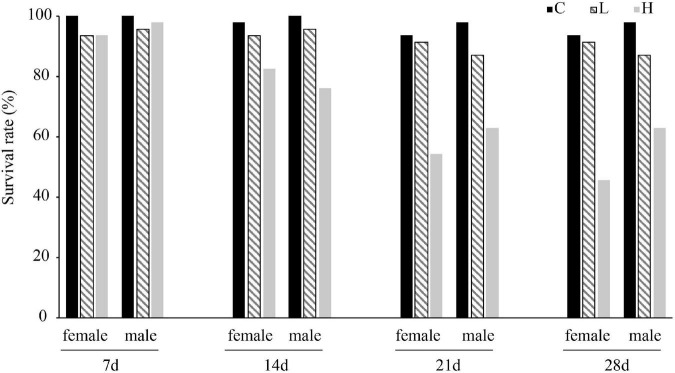
Survival rate of female and male *Pomacea canaliculata* at different temperature and treated time.

After 28 days of temperature stress, the survival rate of male and female *P. canaliculata* was highest in the control group and the lowest in the high-temperature group (Figure 1 and Supplementary Table 1) with one male high temperature groups experienced mass death of snails. Both 35 and 15°C caused the survival rate of snails to decrease, and the negative effect of high temperature on the survival rate of snails was more significant.

Two-way repeated-measures ANOVA showed that stress time had a significant effect on the growth rate of snail length (*F*_1,114_ = 26.907, *P* < 0.001) (Tables 1, 2). Specifically, the interaction of stress time and temperature had a significant effect on both the growth rates (*F*_2,114_ = 29.867, *P* < 0.001) and specific growth rates (*F*_2,114_ = 15.079, *P* < 0.001) of the snails of *P. canaliculata* (Tables 1, 2). The interaction of stress time and sex had a significant effect on the growth rates of snail length and weight gain (*F*_1,114_ = 29.677, *P* < 0.001) (Tables 1, 2), but no significant effect was observed on the specific growth rate. The interaction of stress time, temperature and sex had a significant effect on the growth rate of snail length (*F*_2,114_ = 30.085, *P* < 0.001) (Table 2), but no significant effect was observed on the weight gain rate or the specific growth rate.

**TABLE 1:** Growth rate of snail length, weight gain rate, and specific growth rate of female and male *Pomacea canaliculata* at different temperature and treated time.

Sex	Groups	Growth rate of snail length (%)		Weight gain rate (%)		Specific growth rate (%)	
		0–14 days	14–28 days	0–14 days	14–28 days	0–14 day	14–28 days
Female	Control group (25°C)	0.070	0.063	0.268	0.234	0.017	0.015
	Low-temperature group (15°C)	0.020	0.019	0.065	0.070	0.004	0.005
	High-temperature group (35°C)	0.082	0.054	0.173	0.272	0.011	0.016
Male	Control group (25°C)	0.085	0.069	0.286	0.199	0.018	0.013
	Low-temperature group (15°C)	0.021	0.011	0.102	0.071	0.007	0.005
	High-temperature group (35°C)	0.095	0.080	0.230	0.360	0.014	0.021

**TABLE 2:** Results of two-way repeated measures ANOVA to test the effect of temperature and sex as fixed factors, with treated time as within-subject factor, during different period on growth rate of morphological index.

Factors	Growth rate of snail length	Weight gain rate	Specific growth rate
Treat time	***F***_1,114_ = **26.907, *P*** < **0.001**	*F*_1,114_ = 0.993, *P* = 0.321	*F*_1,114_ = 0.816, *P* = 0.368
Treat time × Temperature	***F***_2,114_ = **29.867, *P*** < **0.001**	*F*_2,114_ = 0.504, *P* = 0.479	***F***_2,114_ = **15.079, *P*** < **0.001**
Treat time × Sex	***F***_1,114_ = **29.677, *P*** < **0.001**	***F***_1,114_ = **14.649, *P*** < **0.001**	*F*_1,114_ = 0.840, *P* = 0.361
Treat time × Temperature × Sex	***F***_2,114_ = **30.085, *P*** < **0.001**	*F*_2,114_ = 0.891, *P* = 0.413	*F*_2,114_ = 0.952, *P* = 0.389

*F* and *P*-values of two-way repeated measures ANOVA. Factors with significant difference are indicated in bold (*P* < 0.05).

### Gut microbial amplicon sequence variant and alpha diversity index

After preliminary filtering, the deduplicated sequences were clustered at 97% similarity level, and 57,537 ASVs were then obtained. According to the species annotation results, bacteria were classified into 54 phyla, 182 classes, 366 orders, 619 families, and 1,370 genera. The rarefaction curves of each group tended to be flat, suggesting that the sequencing results could represent the real species composition in the samples (Supplementary Figure 1).

The Goods coverage index of each group was above 98%, indicating a high level of diversity coverage. The Kruskal–Wallis *H*-test was used to analyze the abundance and diversity of the intestinal microbiota of *P. canaliculata*. Since there was no significant difference in the alpha diversity of intestinal microbiota between female and male *P. canaliculata* (*P* > 0.05), the data of same sex were combined for alpha diversity analysis. The female and male samples were combined for each of the temperature and stress time groups for analysis.

**FIGURE 2:**
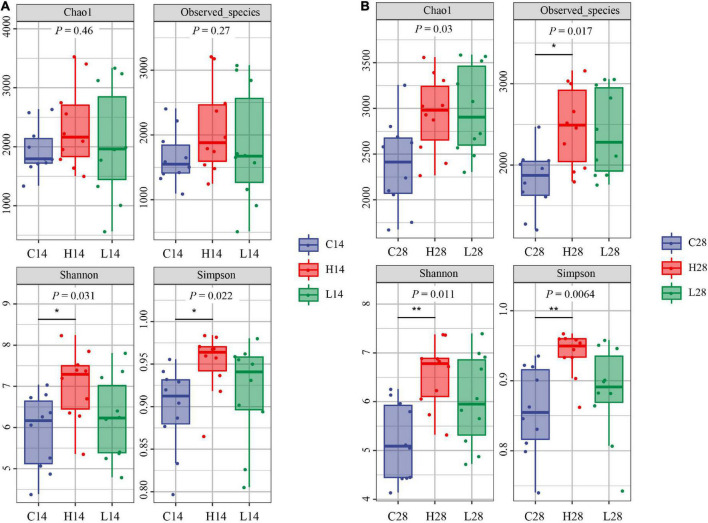
Comparison of intestinal microbiome alpha diversity index of different temperatures on 14 days **(A)** and 28 days **(B)**. C14, H14, and L14 represent the control group, high and low temperature groups treated for 14 days, respectively. C28, H28, and L28 represent the control group, high and low temperature groups treated for 28 days, respectively. *P < 0.05 and <0.01 and **P < 0.01 represented the differences that were significant.

After 14 days of temperature stress treatment, the order of Chao1 index and observed species index from high to low was the low temperature group, high temperature group, and control group (Figure 2A). No significant difference was observed after 14 days of temperature stress treatment. The results of the Chao1 index and observed species index after both 14 and 28 days temperature stress treatment were consistent (Figure 2B). However, the observed species index of the high temperature group was significantly greater than that of the control group after the 28-days temperature stress treatment (*P* < 0.05) (Figure 2B). The order of Shannon index and Simpson index from high to low was: the high temperature group, low temperature group and control group after both 14 and 28 days treatment. The Simpson index in both 14 and 28 days treatment groups was significantly higher than that in the control group (*P* < 0.05) (Figures 2A,B). After 14 or 28 days of temperature stress treatment, the high temperature group had the greatest richness and diversity index for intestinal microbiome, followed by the low temperature group and then the control group (Figures 2A,B).

**FIGURE 3:**
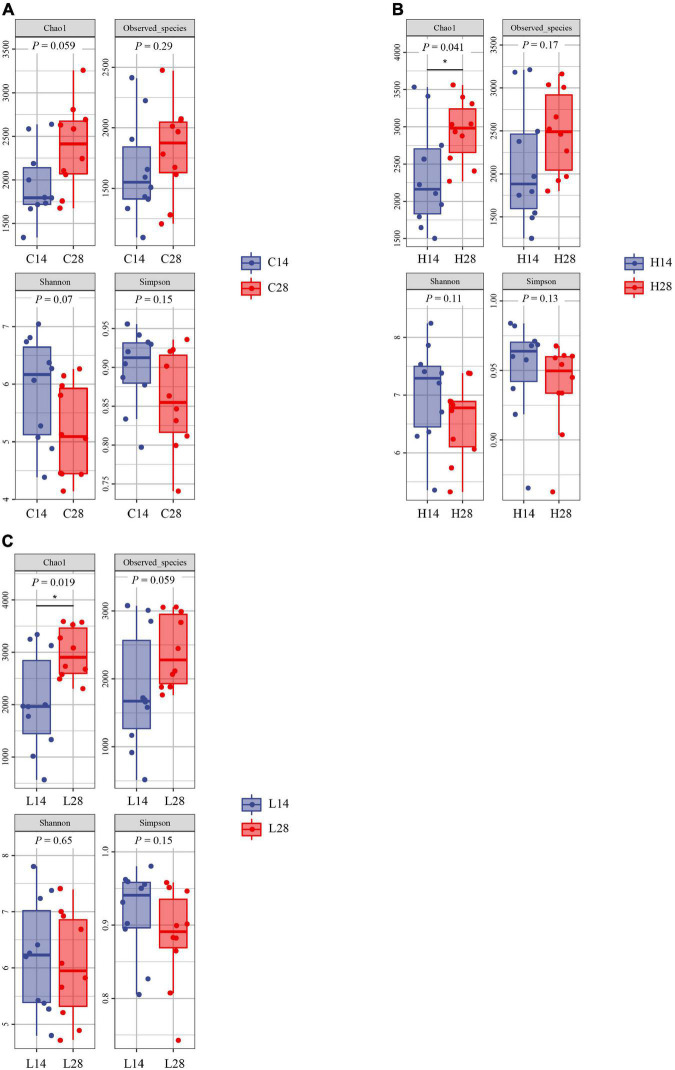
Comparison of intestinal microbiome alpha diversity index of different experimental time points at the control group **(A)**, high temperature group **(B)**, and low temperature group **(C)**. The * denotes a significant difference (*P* < 0.05 and <0.01) between groups.

Both the richness index and diversity index had no significant difference after 14 and 28 days in control groups (Figure 3A). The Chao1 index of 28 days high and low temperature groups was significantly higher than that of the 14 days groups (*P* < 0.05), and no significant difference was observed in other indexes (Figures 3B,C). These results indicated that under the stress of low and high temperature, the abundance of intestinal microbiota of *P. canaliculata* increased significantly with the extension of time (*P* < 0.05).

### Composition of intestinal microbial community of *Pomacea canaliculata* under different temperature stress

**FIGURE 4:**
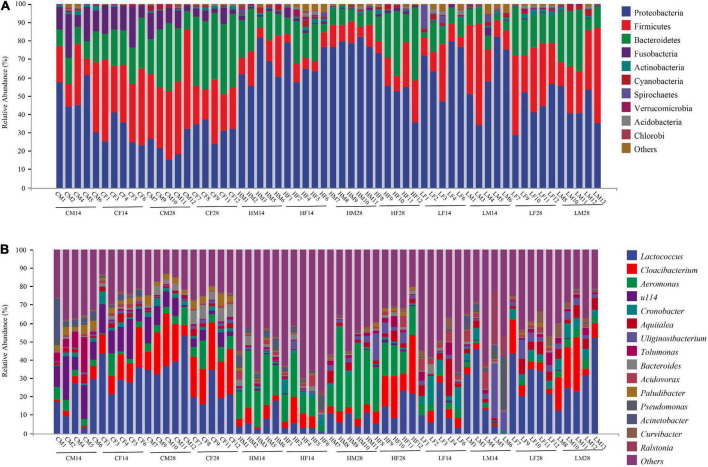
Abundance at the phylum **(A)** and genus **(B)** level of intestine microbiota in *Pomacea canaliculata* treated in different temperatures (mean relative abundance > 1%).

At the phylum level, the intestinal microbiome of *P. canaliculata* in the high-temperature group was mainly composed of Proteobacteria, Firmicutes, Bacteroidetes, and Fusobacteria, with a relative proportion of 32.96, 30.03, 23.68, and 10.80%, respectively. In the control group, the relatively high abundant microbiome included Proteobacteria (66.73%), Bacteroidetes (15.76%), Firmicutes (11.40%), Fusobacteria (1.35%), and Actinobacteria (1.06%). In the low temperature group, the relatively high abundant microbiome included Proteobacteria (54.31%), Bacteroidetes (24.84%), Firmicutes (14.11%), Actinobacteria (1.15%), Cyanobacteria (1.24%), and Spirochetes (1.18%) (Figure 4A).

At the genus level, the dominant genera in the intestinal microbiome of the snails in the high temperature group were *Aeromonas*, *Lactococcus*, and *Cloacibacterium*, accounting for 20.21, 8.24, and 20.24%, respectively. The intestinal microbiome in the control group was mainly composed of *Lactococcus* (27.42%), *Cloacibacterium* (14.00%), *u114* (10.36%), and *Aeromonas* (5.58%). In the low temperature group, the intestinal microbiome of the snail was mainly composed of *Lactococcus* (22.22%), *Cloacibacterium* (7.44%), *Cronobacter* (5.05%), *Aquitalea* (4.71%), *Uliginosibacterium* (4.56%), and *Aeromonas* (2.82%) (Figure 4B).

### Analysis of beta-diversity of the intestinal microbiome of *Pomacea canaliculata* under different temperature stress

**FIGURE 5:**
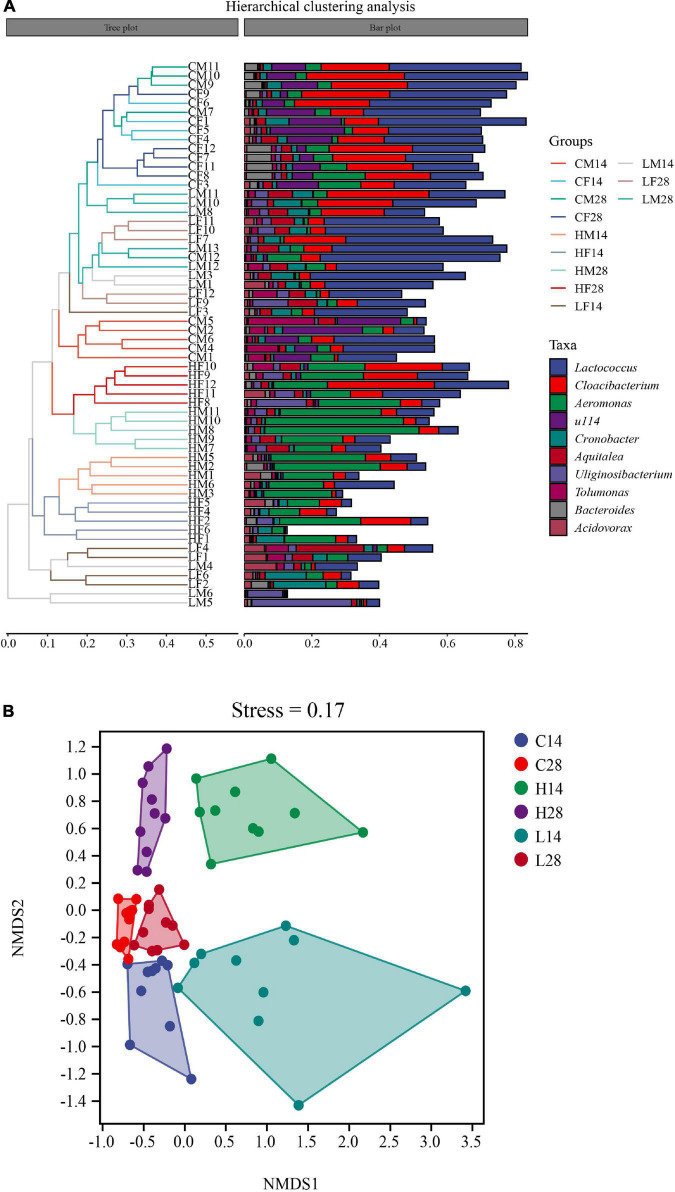
The UPGMA cluster analysis of samples in Bray-Curtis distances **(A)**; NMDS analysis based on Bray-Curtis distances of intestine microbiome **(B)**.

According to the Bray-Curtis distance algorithm, all samples were clustered at the genus level. The results showed that the samples in the high temperature group were first placed into one cluster, while the low temperature and control groups were not divided into two clusters but confusingly classified. The cluster of the high temperature group was further divided into two clusters: the 14 days treatment groups and the 28 days treatment groups (Figure 5A). Because the clustering of the samples in control group and low temperature groups was relatively chaotic, to further study the effects of low temperature and stress time on the intestinal microbiome of *P. canaliculata*, NMDS and ANOSIM analyses were performed based on the Bray-Curtis distance algorithm. The samples were aggregated based on three temperatures and the two stress times, and the results showed that both temperature and stress time had a significant impact on the intestinal microbiome of *P. canaliculata* (*R* = 0.6052, *P* < 0.001) (Figure 5B).

The results of mantel test showed the similar trend that temperature and stress time were significantly correlated with the intestinal microbiome of *P. canaliculata* (*P* < 0.05). Temperature treatment was significantly correlated with intestine microbial communities of *P. canaliculata* in both sexes (*P* < 0.05) (Supplementary Tables 2–4).

### Differences in the intestinal microbial structure of *Pomacea canaliculata* under different temperature stress

**FIGURE 6:**
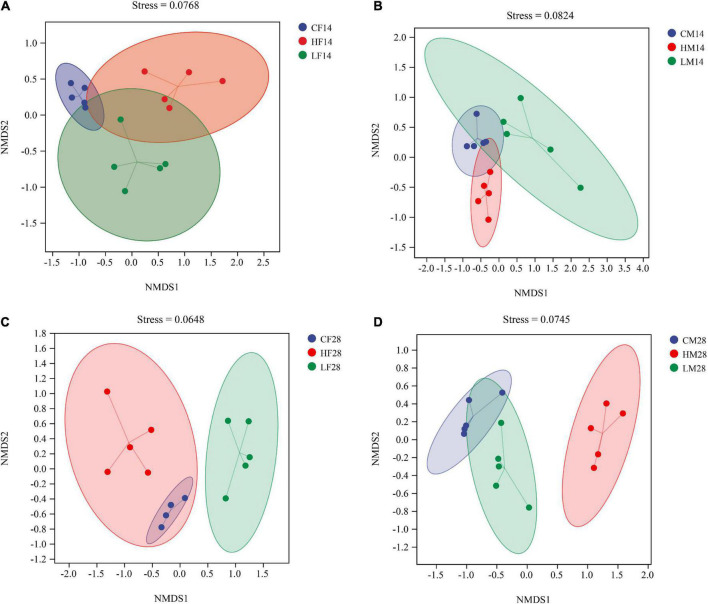
Non-metric multidimensional scaling (NMDS) analysis based on Bray-Curtis distances plots of *Pomacea canaliculata* intestine microbial communities at the different temperature [**(A)** 14 days female; **(B)** 14 days male; **(C)** 28 days female; **(D)** 28 days male].

Non-metric multidimensional scaling and ANOSIM analyses based on the Bray-Curtis distance showed that the structure of the gut microbiota of female snails was significantly different after 14 days stress at different temperatures (*R* = 0.868, *P* < 0.001) (Figure 6A). Similar phenomenon was observed for male snails (*R* = 0.644, *P* < 0.001) (Figure 6B). There were still significant differences in the intestinal community composition of female snails under different temperatures for 28 days (*R* = 0.876, *P* < 0.001) (Figure 6C) and again, similar phenomenon was observed for male snails (*R* = 0.892, *P* < 0.001) (Figure 6D). After both 14 and 28 days of temperature stress treatment, there were significant differences in the gut microbiota structure of *P. canaliculata* under different temperature treatments, indicating that temperature stress had a significant effect on the structure of the intestinal microbiome of *P. canaliculata*.

### Differences in the intestinal microbial structure of *Pomacea canaliculata* with different stress times between different sexes

The NMDS and ANOSIM analysis based on the unweighted-UniFrac distance algorithm showed that the structure of the intestinal microbiome in female *P. canaliculata* varied significantly over time in both high and low temperature groups (*P* < 0.05) (Table 3 and Supplementary Figure 2), indicating that the duration of temperature stress had a significant effect on the intestinal bacteria in female *P. canaliculata*. This result was also supported by NMDS and ANOSIM analysis based on the weighted-UniFrac distance algorithm (Table 3 and Supplementary Figure 2). For males, the duration of temperature stress also had a significant effect on the structure of the gut microbiota (Table 3 and Supplementary Figure 2).

**TABLE 3:** Analysis of similarity (ANOSIM) analyses based on unweighted-UniFrac and weighted-UniFrac distances of *Pomacea canaliculata* intestine microbial communities at the different experimental time points.

Groups	Unweighted-uniFrac distance	Weighted-uniFrac distance
HF14 and HF28	*R* = 0.908, *P* = 0.008	*R* = 0.656, *P* = 0.007
CF14 and CF28	*R* = 1.000, *P* = 0.008	*R* = 0.868, *P* = 0.011
LF14 and LF28	*R* = 0.328, *P* = 0.010	*R* = 0.656, *P* = 0.010
HM14 and HM28	*R* = 0.928, *P* = 0.007	*R* = 0.580, *P* = 0.010
CM14 and CM28	*R* = 0.868, *P* = 0.009	*R* = 0.708, *P* = 0.008
LM14 and LM28	*R* = 0.592, *P* = 0.004	*R* = 0.336, *P* = 0.009

The ANOSIM analysis based on the Bray-Curtis distance algorithm also showed significant difference in the intestinal microbiome between female and male *P. canaliculata*. Significant difference was also observed between 14 and 28 days treatment groups and between high and low temperature groups (*R* > 0.5, *P* < 0.05) (Figure 6).

### Differential analysis of the gut microbiome at different temperatures, different stress times, and different sexes

**FIGURE 7:**
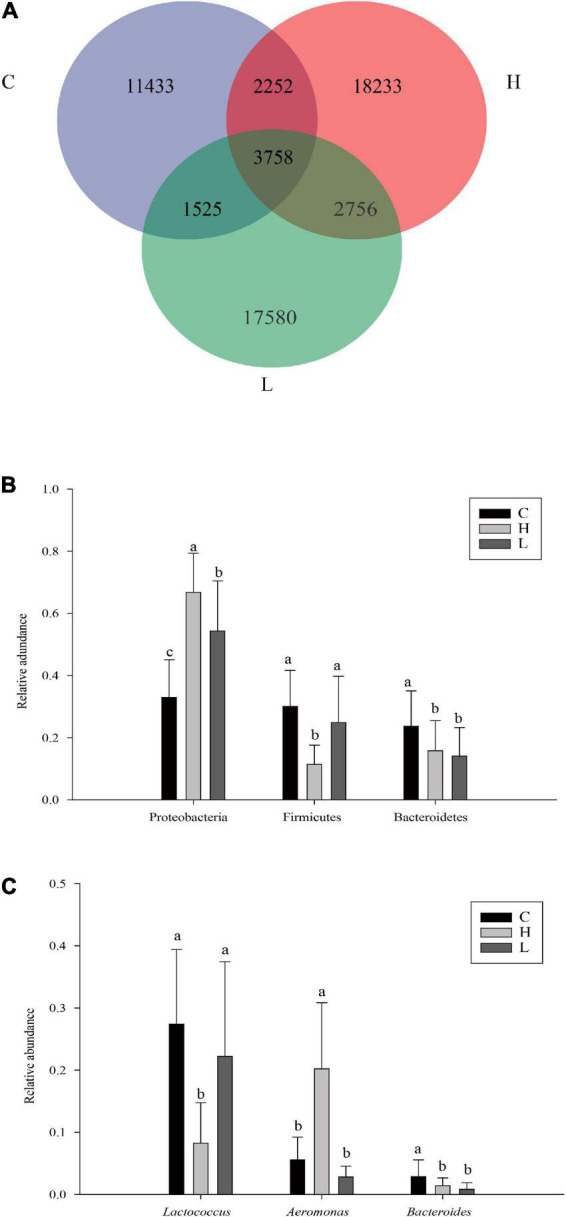
Venn analysis of samples from different temperature **(A)**. The relative abundances of dominant phylum **(B)** and genus **(C)** of *Pomacea canaliculata* intestine microbiome at different temperature group (Duncan’s post hoc test, a = 0.05; a>b>c). The C, H, and L represented the control group, high and low temperature groups treated for 14 and 28 days, respectively.

It can be seen from the Venn diagram that the high, low temperature and control groups had a total of 3,758 ASVs, of which the high temperature group had 18,233 ASVs (13.92%), the low temperature group had 17,580 ASVs (14.67%), and the control group had 11,433 ASVs (19.81%) (Figure 7A).

At the phylum level, Proteobacteria, Firmicutes, and Bacteroidetes were the dominant phyla shared by these groups. The relative abundance of important dominant bacterial phyla in the high, low temperature and control groups was compared based on one-way analysis of variance. The results showed that the relative abundance of Proteobacteria in the high temperature group was the largest (66.73%), which was significantly higher than that of the low temperature group (54.31%) and control group (32.96%) (*F*_2,57_ = 31.05, *P* < 0.001). Firmicutes also had significant differences among different temperature groups, and its relative abundance in the high temperature group (11.40%) was significantly lower than that in the low temperature (24.84%) and control groups (30.03%) (*F*_2,57_ = 14.04, *P* < 0.001). Furthermore, the relative abundance of Bacteroidetes decreased sequentially in the control (23.68%), high (15.76%), and low temperature (14.11%) groups. The relative abundance of Bacteroidetes in the control group was significantly greater than that in the high and low temperature groups (*F*_2,57_ = 5.14, *P* = 0.0089) (Figure 7B).

At the genus level, *Lactococcus*, *Aeromonas*, and *Bacteroides* were the dominant genera shared by the high, low temperature and control groups. The relative abundance of *Lactococcus* in the high temperature group (8.24%) was significantly lower than that in the low temperature (22.22%) and control (27.42%) groups (*F*_2,57_ = 14.14, *P* < 0.001). The relative abundance of *Aeromonas* in the high temperature group (20.21%) was significantly higher than that in the low temperature (2.82%) and control (5.58%) groups (*F*_2,57_ = 40.43, *P* < 0.001). Additionally, the relative abundance of *Bacteroides* decreased successively in the high, low temperature and control groups, and their relative abundances were 1.41, 0.83, and 2.84%, respectively. The relative abundance of *Bacteroides* in the high and low temperature groups was significantly lower than in the control group (*F*_2,57_ = 6.38, *P* = 0.003) (Figure 7C).

**FIGURE 8:**
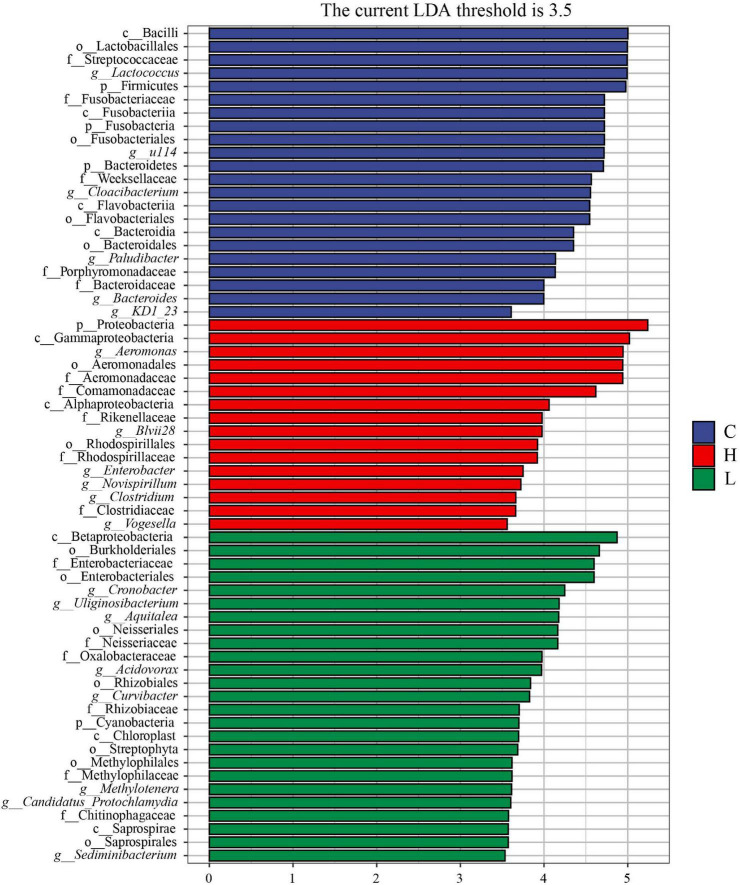
LEfSe analysis of *Pomacea canaliculata* intestinal microbiota composition under different temperature. The c, g, f, o, and p represented class, genus, family, order, phylum, respectively.

To further explore the potential biomarkers with statistically significant differences between the high, low temperature and control groups, LEfSe analysis was performed on the samples with different stress times and sex (| LDA| > 3.5). There were one phylum, two classes, two orders, four families, and six genera in the microbiota with different abundances in the high temperature group, and Proteobacteria, Rikenellaceae, Rhodospirillaceae, *Aeromonas*, *Blvii28*, *Enterobacter*, *Novispirillum*, and *Vogesella* were significantly enriched (Figure 8). There were one phylum, three classes, seven orders, six families, and eight genera in the low temperature group, and Cyanobacteria, Neisseriales, Enterobacteriaceae, Neisseriaceae, Oxalobacteraceae, *Cronobacter*, *Uliginosibacterium*, *Aquitalea*, *Acidovorax*, and *Methylotenera* were significantly enriched (Figure 8). There were three phyla, four classes, four orders, five families, and six genera in control group, and Firmicutes, Fusobacteria, Bacteroidetes, Streptococcaceae, Fusobacteriaceae, Porphyromonadaceae, Bacteroidaceae, *Lactococcus*, *Cloacibacterium*, and *Bacteroides* were significantly enriched (Figure 8).

Furthermore, there were two phyla, four classes, five orders, five families, and four genera with different abundances in the intestinal microbiome of *P. canaliculata* under 14-days low temperature stress, including Proteobacteria, Fusobacteria, Lachnospiraceae, Bacteriovoracaceae, *Ralstonia*, *Pseudomonas*, and *Acidovorax*. There were two phyla, two classes, four orders, four families, and four genera significantly enriched in the 28-days low temperature stress group, including Bacteroidetes and Firmicutes, *Lactococcus*, and *Chryseobacterium* (Supplementary Figure 3A).

There were three classes, seven orders, 10 families, and seven genera with different abundances in the 14-days high temperature stress group, including Spirochetes, Comamonadaceae, Rhodospirillaceae, Oxalobacteraceae, Lachnospiraceae, *Pseudomonas*, *Shewanella*, *Acinetobacter*, *Acidovorax*, and *Flavobacterium*. There were one class, three orders, three families, and six genera with significant differences in abundance in the 28-days high temperature stress group, including Streptococcaceae, Neisseriaceae, *Lactococcus*, *Enterobacter*, and *Aquitalea* (Supplementary Figure 3B).

In the 28-days low temperature group (L28), Actinobacteria, Acidobacteria, and *Comamonas* were significantly enriched in the female samples, while *Aeromonas*, *Cronobacter*, and *Enterobacter* were significantly enriched in the male samples (Supplementary Figure 4A). In the 28-days high temperature group (H28), Bacteroidetes, Fusobacteria, and *Bacteroides* were significantly enriched in the female samples, while Proteobacteria, Enterobacteriaceae, and *Sulfurospirillum* were significantly enriched in the male samples (Supplementary Figure 4B). In the 28-days control group (C28), Bacteroidetes, Proteobacteria, *Bacteroides*, *Enterobacter*, and *Aquitalea* were significantly enriched in the female samples, while Fusobacteria and *Lactococcus* were significantly enriched in the male samples (Supplementary Figure 4C).

## Discussion

The intestinal microbiome is involved in various functions, such as immune regulation, gastrointestinal motility, and energy metabolism regulation (Paaijmans et al., 2013). In this study, we used the Illumina Novaseq sequencing technology of the 16S rRNA gene to investigate the response of the intestinal microbiome of *P. canaliculata* to temperature stress. The results showed that the alpha diversity and community structure of the intestinal microbiome of *P. canaliculata* changed significantly under high temperature (35°C) and low temperature (15°C) stress.

### Effect of temperature stress on the growth and development of *Pomacea canaliculata*

Previous studies have shown that the survival rate of *P. canaliculata* is negatively correlated with temperature, and the survival rate is the lowest at 35°C (Seuffert and Martín, 2013). In the high temperature range (25, 30, and 35°C), the survival rate of *P. canaliculata* decreased with increasing temperature, while in the low temperature range (0 and 15°C), the survival rate increased with decreasing temperature (Seuffert and Martín, 2013). The present study showed that the survival rate of *P. canaliculata* was the lowest in the high temperature group (54.35%), followed by low temperature group and control group (Figure 1 and Supplementary Table 1). In addition, after 21 days of high temperature stress, the survival rates of female and male *P. canaliculata* were no longer significantly reduced, indicating that *P. canaliculata* may have gradually adapted to the high temperature environment over a long-term treatment.

This study showed that the growth rate of snail length in the control group was significantly greater than that in the high and low temperature groups, and the growth rate and specific growth rate for snail weight in the high temperature group and control group were significantly greater than those in the low temperature group, (Tables 1, 2), consistent with previous studies (Seuffert et al., 2010). In addition, the increased growth rate of snail length of female *P. canaliculata* in the low temperature group was significantly greater than that of male *P. canaliculata* (Tables 1, 2), which may be closely related to the higher plant digestibility and foraging rate of female snails (Tamburi and Martín, 2009; Xu et al., 2011).

### Effect of temperature on the intestinal microbiome of *Pomacea canaliculata*

The structural composition of the gut microbiota of aquatic organisms is affected by ambient temperature (Hagi et al., 2004; Pierce et al., 2016). The survival rate of the mussel *Mytilus galloprovincialis* significantly decreased under high temperature stress at 27°C, while the diversity of its gut microbiota increased significantly (Li et al., 2019). In our study, both high and low temperature stress led to an increase in the diversity index of the intestinal microbiome of *P. canaliculata* (Figures 2A,B). It is important to note that the increased diversity of the intestinal microbiome is often beneficial for animals to cope with changes in the external environment and maintain the dynamic balance of the intestinal microenvironment (Sommer et al., 2017), indicating that the increase in the alpha diversity index may be related to the adaptation to the stress environment of *P. canaliculata*.

The gut microbiome of aquatic animals is quite susceptible to temperature (Sepulveda and Moeller, 2020). The gut bacterial community structure of *Mytilus coruscus* was significantly different at 27 and 31°C, and the main bacterial species were strongly affected by temperature (Li et al., 2018). When comparing the gut microbiota of *Chanos chanos* during 3 weeks of control (26°C) and elevated temperature (33°C) treatments, significant difference was observed soon after the temperature was increased, but tended to become similar, and no significant difference was observed at the end of the experiment. Changes in the gut microbiota due to heat stress can contribute to host adaptation to high temperatures (Hassenrück et al., 2020). In this study, temperature also had a significant effect on the gut microbiota structure of *P. canaliculata*. The hierarchical clustering analysis of samples based on the Bray-Curtis distance algorithm showed that the high temperature group was clustered alone and separated from the low temperature and control groups (Figure 5A). While there was no obvious separation between the low temperature and control group, which may be due to the low temperature stress of 15°C having no obvious effect on *P. canaliculata* (Pan et al., 2008). However, there were significant differences in the gut microbiota structure of *P. canaliculata* after 14 days (Figures 6A,B) and 28 days (Figures 6C,D) of stress at different temperatures. Similar results reported in the sea urchin (*Echinometra* sp. *EZ*) with gut microbiota varying across thermally variable habitats and displaying temporal shifts correlated with temperature (Ketchum et al., 2021).

Although temperature caused significant changes in the gut microbiota structure of *P. canaliculata*, the composition of its core microbiota was not greatly affected, and only their relative abundances were changed. The dominant core microbiota at the phylum level in all samples included Proteobacteria, Bacteroidetes, and Firmicutes (Figure 4A). The dominant core microbiota at the genus level included *Aeromonas*, *Lactococcus*, and *Cloacibacterium* (Figure 4B), suggesting that the core microbiota play a critical role in maintaining the basic physiological functions of the snail.

At the phylum level, most bacteria in Proteobacteria are facultative anaerobes and primarily pathogenic bacteria, which may lead to intestinal inflammation (Lupp et al., 2007). The increased abundance of Proteobacteria is often closely related to energy imbalance and instability in the host gut microbiota, which usually occur in the process of host metabolic disorders (Shin et al., 2015). In a study of the insect gut microbiota, elevated temperature was found to be associated with an increase in the relative abundance of Proteobacteria (Sepulveda and Moeller, 2020). For example, in the intestinal microbiota of *Porcellio scaber*, the relative abundance of Actinobacteria decreased with increasing temperature, and the relative abundance of Proteobacteria increased with increasing temperature (Horváthová et al., 2019). In contrast to insects, a positive correlation between the relative abundance of Proteobacteria and temperature has rarely been observed in mollusks (Sepulveda and Moeller, 2020). In this study, the relative abundance of Proteobacteria in the high temperature group was significantly greater than that in the low temperature group, and the relative abundance of Proteobacteria in the low temperature group was significantly greater than that in the control group (Figure 7B), indicating that the high and low temperature environments can lead to a stress response in *P. canaliculata*, resulting in the enrichment of pathogenic bacteria and a change in the intestinal microbiota structure. Bacteroidetes are mainly responsible for promoting carbohydrate fermentation and are involved in a variety of functions, such as polysaccharide, bile acid, and steroid metabolism as well as the maintenance of the intestinal microbiota balance (Sears, 2005). Inflammation of the gut is often accompanied by a significant reduction in the abundance of Bacteroidetes (Scaldaferri et al., 2013). This result was also confirmed in the study of the gut microbiota of the rainbow trout (*Oncorhynchus mykiss*) (Zhou et al., 2022) and mussel (*M. galloprovincialis*) (Li et al., 2019) under acute temperature stress. The relative abundance of Bacteroidetes in the high and low temperature groups in this study was significantly lower than that in the control group (Figure 7C), indicating that high and low temperature had adverse effects on the health status of *P. canaliculata* and could affect the maintenance of intestinal homeostasis and the development of host immunity (Colston and Jackson, 2016). Previous studies showed that increasing temperature tends to reduce the abundance of Firmicutes (Bestion et al., 2017; Zhu et al., 2019). A similar effect was observed in *Lithobates pipiens*, where the relative abundance of Firmicutes decreased significantly when the temperature increased from 18 to 28°C (Kohl et al., 2016). The mechanism by which the relative abundance of Firmicutes is negatively correlated with temperature is still unclear. It is speculated that the animal host may maintain the number of Firmicutes in the gut through active metabolism. Therefore, a metabolic disorder in the host caused by heat stress may lead to the prevalence of this phylum’s reduction (Sepulveda and Moeller, 2020). In this study, the relative abundance of Firmicutes in the high temperature group was significantly lower than that in the low temperature group and control group (Figure 7B), which is similar to previous conclusions and indicates that the sharp increase in temperature is likely to inhibit the growth of Firmicutes in the intestinal tract, causing the occurrence of inflammation and resulting in the death of *P. canaliculata*.

*Lactococcus* was found to be one of the dominant genera in the intestinal lactic acid microbiota of *Cornu aspersum* and *Oncomelania hupensis* (Koleva et al., 2014; Hao et al., 2020). Furthermore, the abundance of lactic acid bacteria (especially *Lactococcus*) in the gut microbiota of *O. mykiss* reduced significantly after 6 weeks of treatment under heat stress at 18°C (Huyben et al., 2018). This result is consistent with our results in this study that the relative abundance of *Lactococcus* in the high temperature group was significantly lower than that in the low temperature group and control group, presumably due to the increased temperature inhibiting the growth of *Lactococcus* (Figure 7C). Additionally, *Clostridium* has the ability to degrade cellulose and cellobiose (Kohl et al., 2016). In this study, this genus was significantly enriched in the high temperature group, which may play an important role in promoting the digestion and absorption of food by the host.

Temperature-induced changes in the gut microbiota may also lead to increase in pathogenic microbiota (Fontaine et al., 2018). *Aeromonas* is a gram-negative facultative anaerobic bacterium, most of which have been identified as pathogenic to some organisms, including fish (Dar et al., 2016) and shellfish (Chen J. S. et al., 2021; De Silva et al., 2021). The relative abundance of *Aeromonas* in the high temperature group was significantly higher than that in the low temperature and the control groups (Figure 7C). *Enterobacter* was also significantly enriched in the high temperature group. When the temperature changes sharply, the intestinal microbiota is disordered, and the abundance of pathogenic bacteria increases, which, to a certain extent, can explain the higher mortality rate of the snails in the high temperature group. Massive proliferation of opportunistic pathogens such as Vibrio and Arcobacter under heat stress conditions could increase the susceptibility of the host to disease and result in the mass death of mussels (Li et al., 2019).

### Effect of temperature stress time on the intestinal microbiome of *Pomacea canaliculata*

The plasticity of gut microbial communities further helps species respond to new ecological challenges and adapt to new environments (Alberdi et al., 2016). Studies have shown that the gut microbiota of the invasive bullfrog (*Lithobates catesbeianus*) is more plastic than that of the green frog (*Lithobates clamitans*). Bullfrogs gut microbiota changes after only hours of warmer temperatures, whereas in green frogs this change occurs significantly after days (Fontaine and Kohl, 2020).

There were significant differences in the gut microbiota structure of *P. canaliculata* after 14 days of stress at different temperatures, and the differences in the structure of the gut microbiota were more obvious after 28 days of stress. According to the results of the LEfSe analysis, many pathogenic bacteria including *Pseudomonas*, *Acinetobacter*, *Shewanella*, and *Flavobacterium* were enriched after 14 days of temperature stress, which were significantly enriched in the high temperature group (H14) (Supplementary Figure 3B). Pathogenic bacteria such as Proteobacteria, *Pseudomonas*, and Bacteriovoracaceae were significantly enriched in the low temperature group (L14) (Supplementary Figure 3A). Some strains of *Flavobacterium* sp. have pathogenic effects on aquatic animals such as freshwater fish and marine fish (Bernardet, 2011), and the mortality rate of diseased fish can be as high as 100%, with symptoms such as surface ulcers, gill rot, and tissue necrosis.

After 28-days of temperature stress, the number of pathogenic bacteria decreased, and many beneficial bacteria were enriched, such as *Lactococcus*, Bacteroidetes, Firmicutes, etc. As common probiotics, they were significantly enriched in the later stages of the high and low temperature stress groups (H28 and L28) (Supplementary Figures 3A,B). Bacteroidetes are mainly involved in the degradation of carbohydrates, metabolism of sugars, and metabolism of substances such as bile acids, which are closely related to host health (Ghanbari et al., 2015). Firmicutes contribute to the maintenance of intestinal homeostasis and the development of host immunity (Colston and Jackson, 2016), and are more efficient than Bacteroidetes as an energy source, promoting caloric absorption and nutrient transport, thereby leading to weight gain (Zhu et al., 2021). The decrease of pathogenic bacteria and enrichment of a large number of probiotics in the later stage of temperature stress were highly consistent with the significant increase in the abundance of the intestinal microbiota of *P. canaliculata* over time. This finding indicates that the intestinal microbiome may have begun to adapt to the stress environment at the later stage of temperature stress. A similar trend was observed in the morphological analysis of *P. canaliculata*. After 21 days of high temperature stress, the survival rates of both female and male *P. canaliculata* were no longer significantly reduced, indicating that the surviving *P. canaliculata* may have gradually adapted to the high temperature environment under long-term treatment. Whether this is due to changes in the host’s physiological growth state caused by changes in the gut microbiota or the effect of the gut microbiota response to temperature on the health of the host remains unclear. Further studies are needed to clarify the relationship between the host and gut microbiome as it pertains to temperature stress response.

### Differences in the intestinal microbiome between male and female *Pomacea canaliculata*

Previous studies have shown that sex is also one of the important factors affecting the gut microbiome of animals. The gut microbiota diversity of the male large-finned mudskipper (*Periophthalmus magnuspinnatus*) was significantly higher than that of females (Ma et al., 2018). Also, the microbial communities of male and female blackhead minnows (*Pimephales promelas*) were significantly segregated according to beta diversity metrics (Debofsky et al., 2020). When comparing with the effects of temperature and stress time on the intestinal microbiome of *P. canaliculata*, the differences in the intestinal microbiota between male and female *P. canaliculata* were smaller, but still significant. There was no significant difference in the alpha diversity index of the intestinal microbiota between female and male *P. canaliculata* (*P* > 0.05), but the ANOSIM analysis results based on the Bray-Curtis distance algorithm still showed significant differences between females and males in the high, low temperature and control groups.

According to the LEfSe analysis results, *Enterobacter* and *Aeromonas* were significantly enriched in male *P. canaliculata* after 28 days of low temperature stress (Supplementary Figure 4A). Proteobacteria and Enterobacteriaceae were also significantly enriched in male *P. canaliculata* after 28 days of high temperature stress (Supplementary Figure 4B). Proteobacteria, Enterobacteriaceae, *Enterobacter*, and *Aeromonas* are all opportunistic pathogens. The increased numbers of these opportunistic pathogens greatly increase the risk of bacterial infection in male *P. canaliculata*, which may be a reason why male snails are more susceptible to heat stress (Song et al., 2014). In addition, Bacteroidetes, Fusobacterium, *Bacteroides* and other species closely related to cellulose decomposition bacteria were significantly enriched in female *P. canaliculata* in high temperature and control groups, which may be closely related to the higher plant digestion ability and foraging rate of female *P. canaliculata* (Tamburi and Martín, 2009; Xu et al., 2011).

## Conclusion

In this study, 16S rRNA high-throughput sequencing technology was used to study the growth, development and intestinal microbiome of *P. canaliculata*. The results showed that long-term high and low temperature stress had negative effects on the health status of *P. canaliculata*. Both high and low temperature stress had a significant impact on the intestinal microbiome of *P. canaliculata*, resulting in a significant increase in diversity and enrichment of pathogenic bacteria. The NMDS analysis showed that after 14 days of temperature stress, the intestinal microbiome structure of *P. canaliculata* was significantly changed to adapt to adverse environments including prolonged stress time, increased abundance of beneficial bacteria increased, and decreased opportunistic pathogens.

## Data availability statement

The datasets presented in this study can be found in online repositories. The names of the repository/repositories and accession number(s) can be found in NCBI BioProject PRJNA843708.

## Ethics statement

The animal study was reviewed and approved by the Animal Research Ethics Committee of Nanjing Normal University (Permit No. IACUC-20220267).

## Author contributions

LC conceived the study. SL, ZQ, SG, and WS performed the experiments. SL and ZQ analyzed the data and drafted the manuscript under guidance of LC and HL. XL, HL, and LC revised the manuscript. All authors reviewed the manuscript.

## Footnotes

1
https://docs.qiime2.org/2019.4/tutorials/

2
http://www.arb-silva.de

## Supplementary material

Supplementary Figure 1 Rarefaction Curve for all sample. HF14, LF14, CF14, HM14, LM14, and CM14 represent the female high temperature group, female low temperature group, female control group, male high temperature group, male low temperature group, and male control group treated for 14 days, respectively. HF28, LF28, CF28, HM28, LM28, and CM28 represent the female high temperature group, female low temperature group, female control group, male high temperature group, male low temperature group, and male control group treated for 28 days, respectively.

Supplementary Figure 2 Non-metric multidimensional scaling (NMDS) analysis based on **(A,C,E)** unweighted-UniFrac distances and **(B,D,F)** weighted-UniFrac distances plots of female *Pomacea canaliculata* intestine microbial communities at the different stress time in control group, high and low temperature groups, respectively. NMDS analysis based on **(G,I,K)** unweighted-UniFrac distances and **(H,J,L)** weighted-UniFrac distances plots of male *P. canaliculata* intestine microbial communities at the different stress time in control group, high and low temperature groups, respectively.

Supplementary Figure 3 LEfSe analysis of *Pomacea canaliculata* intestinal microbiota composition at different experimental time points under low **(A)** and high temperature **(B)** stress.

Supplementary Figure 4 LEfSe analysis of female and male *Pomacea canaliculata* intestine microbiome under **(A)** high temperature, **(B)** low temperature, and **(C)** control.

Supplementary Table 1 Survival rate of female and male *Pomacea canaliculata* at different temperature and treated time.

Supplementary Table 2 Mantel tests for the correlations between *Pomacea canaliculata* intestine microbiota and the different temperature groups using Spearman’s coefficients. The C, H, and L represented the control group, high and low temperature groups treated for 14 and 28 days, respectively. C14, H14, and L14 represent the control group, high and low temperature groups treated for 14 days, respectively. C28, H28, and L28 represent the control group, high and low temperature groups treated for 28 days, respectively.

Supplementary Table 3 Mantel tests for the correlations between *Pomacea canaliculata* intestine microbiota and the different experimental time points using Spearman’s coefficients. C14, H14, and L14 represent the control group, high and low temperature groups treated for 14 days, respectively. C28, H28, and L28 represent the control group, high and low temperature groups treated for 28 days, respectively.

Supplementary Table 4 Mantel tests for the correlations between female, male *Pomacea canaliculata* intestine microbiota and the different temperature groups using Spearman’s coefficients. CF, HF, and LF represent the female control group, female high temperature group and female low temperature group treated for 14 and 28 days, respectively. CM, HM, and LM represent the male control group, male high temperature group and male low temperature group treated for 14 and 28 days, respectively.
