# Supplementary figures and images for: Effect of long-term temperature stress on the intestinal microbiome of an invasive snail
Source: Front Microbiol. 2022 Aug 29;13:961502. doi: 10.3389/fmicb.2022.961502 (PMC9465035; doi:10.3389/fmicb.2022.961502)

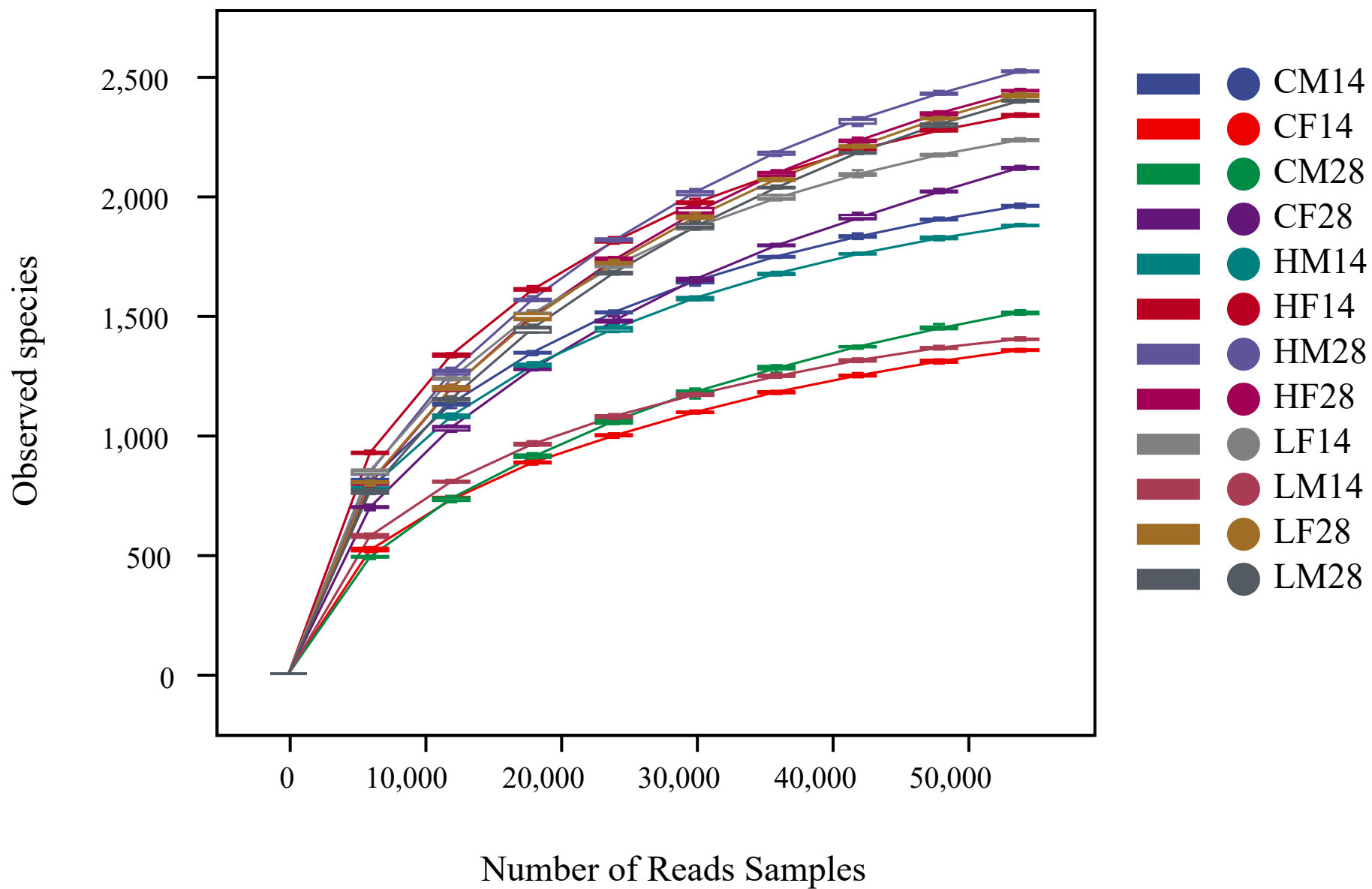

Supplement: Supplementary Figure 1 — Rarefaction Curve for all sample. HF14, LF14, CF14, HM14, LM14, and CM14 represent the female high temperature group, female low temperature group, female control group, male high temperature group, male low temperature group, and male control group treated for 14 days, respectively. HF28, LF28, CF28, HM28, LM28, and CM28 represent the female high temperature group, female low temperature group, female control group, male high temperature group, male low temperature group, and male control group treated for 28 days, respectively. [file Image_1.pdf]

A

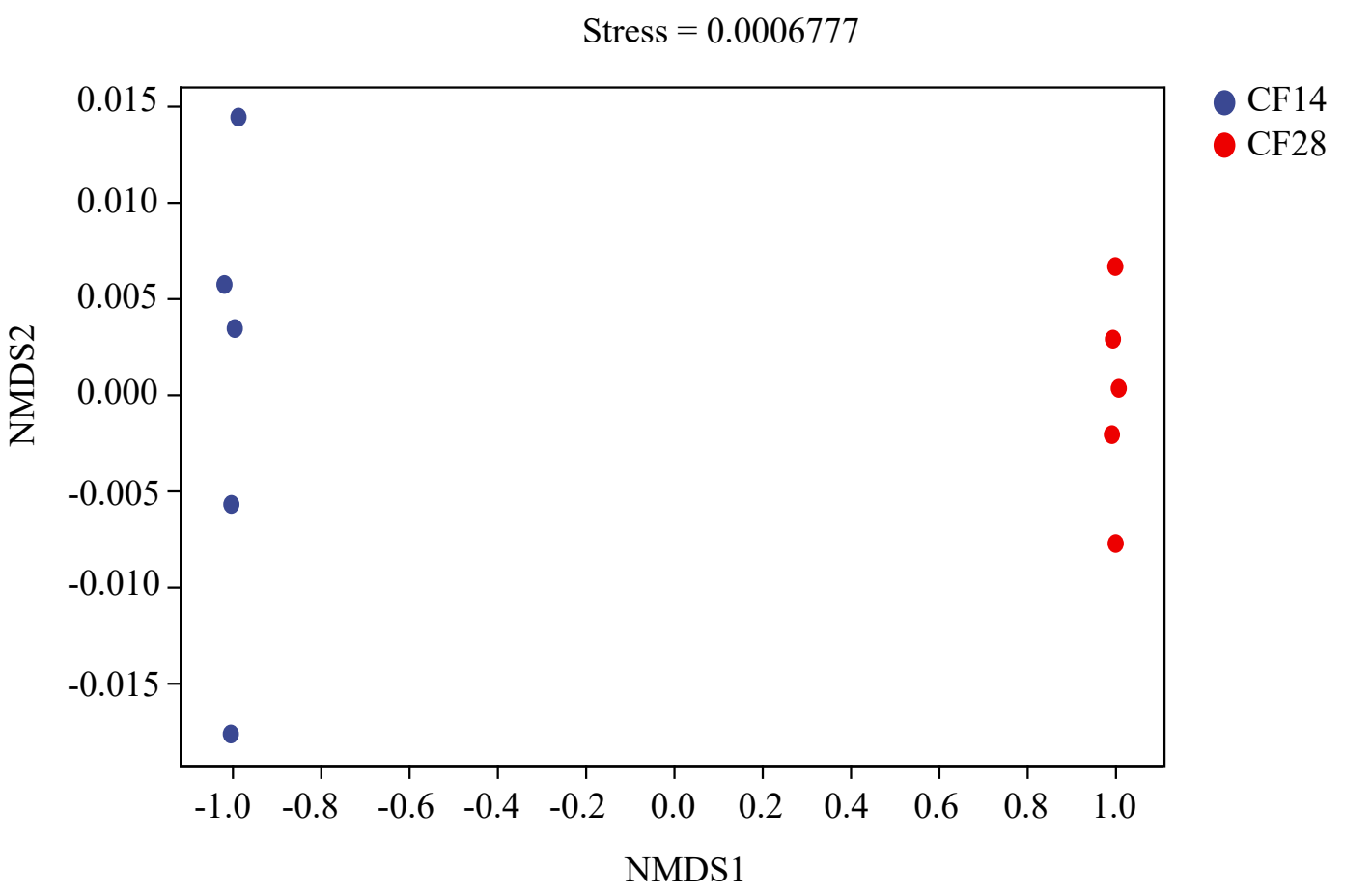

B

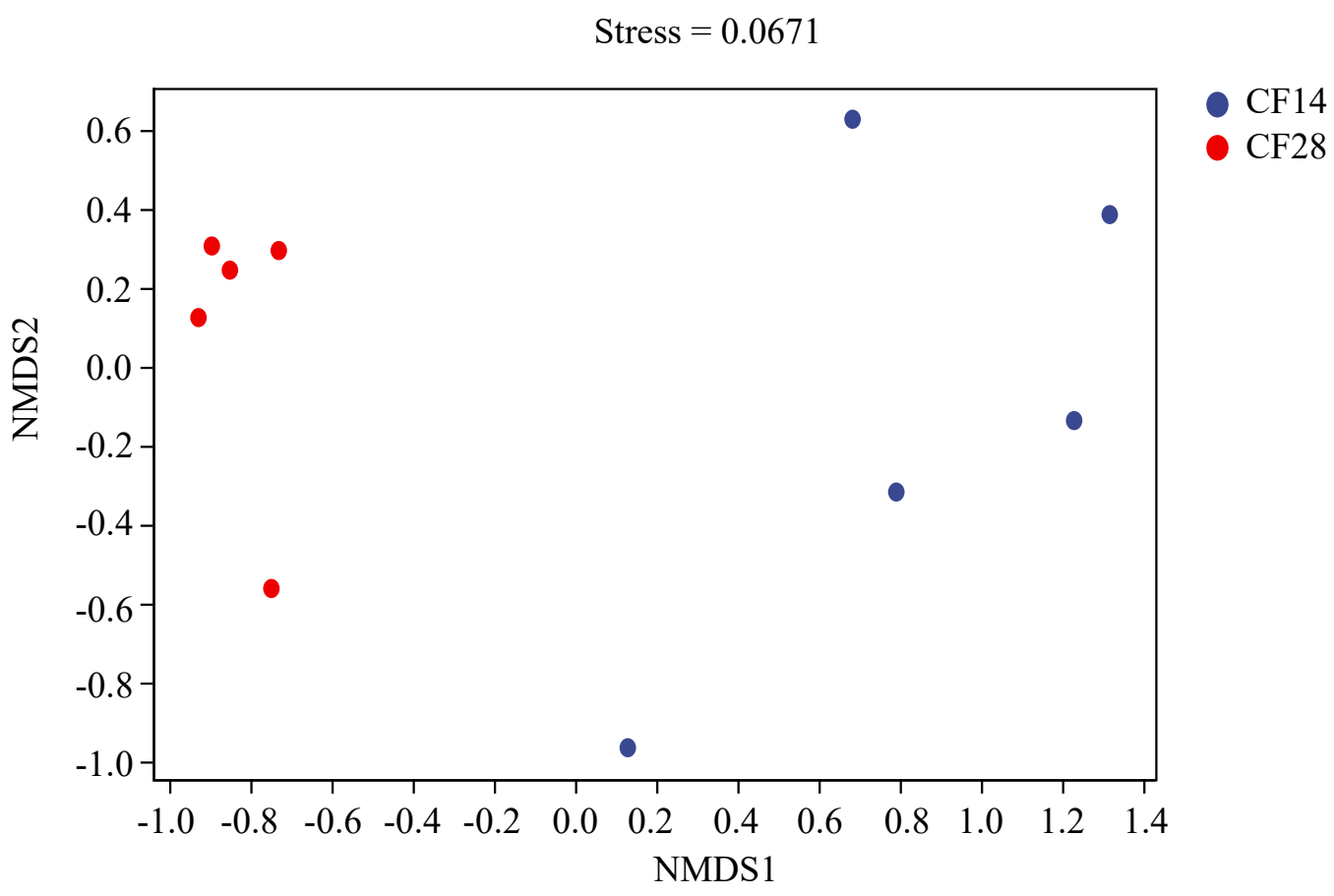

C

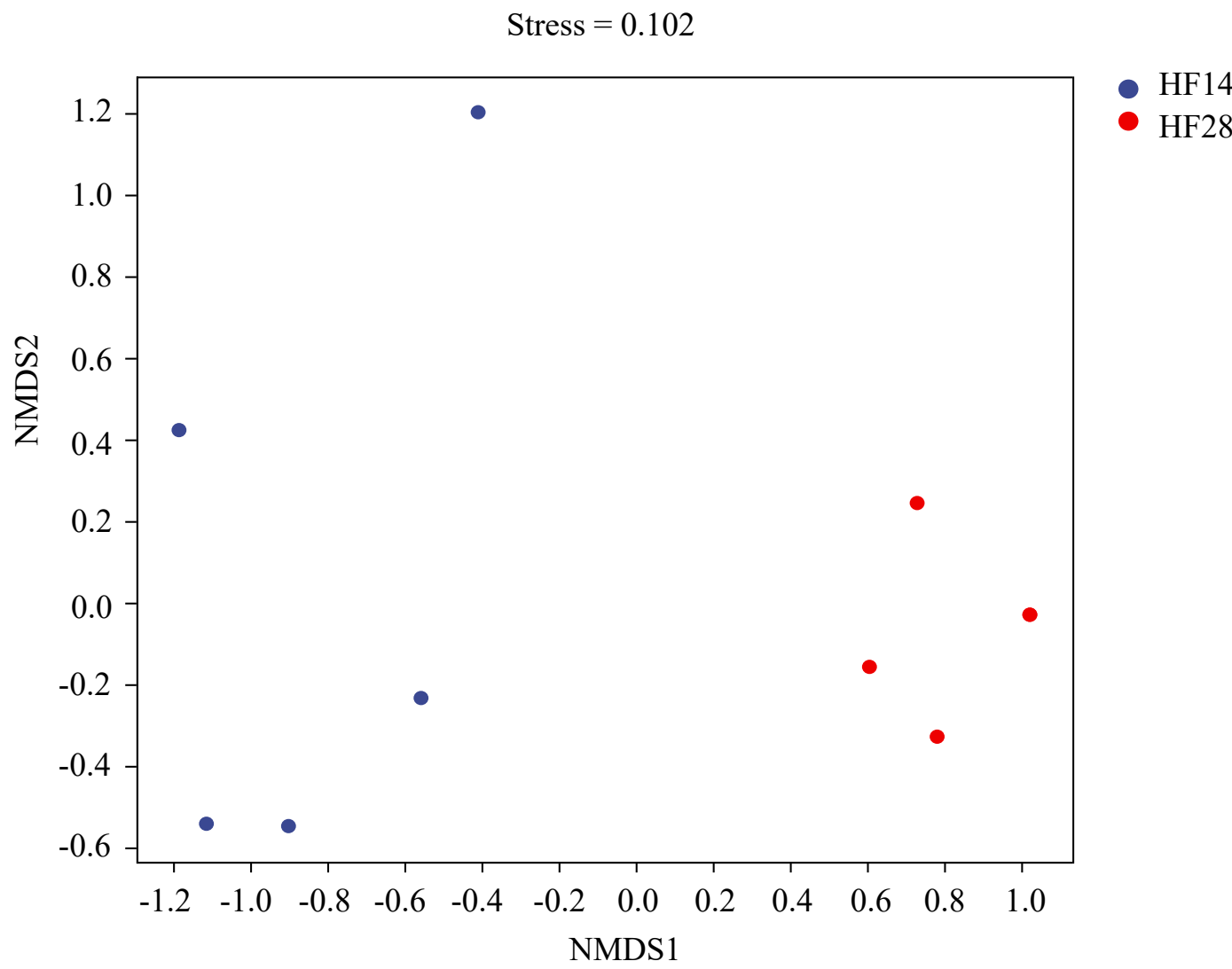

D

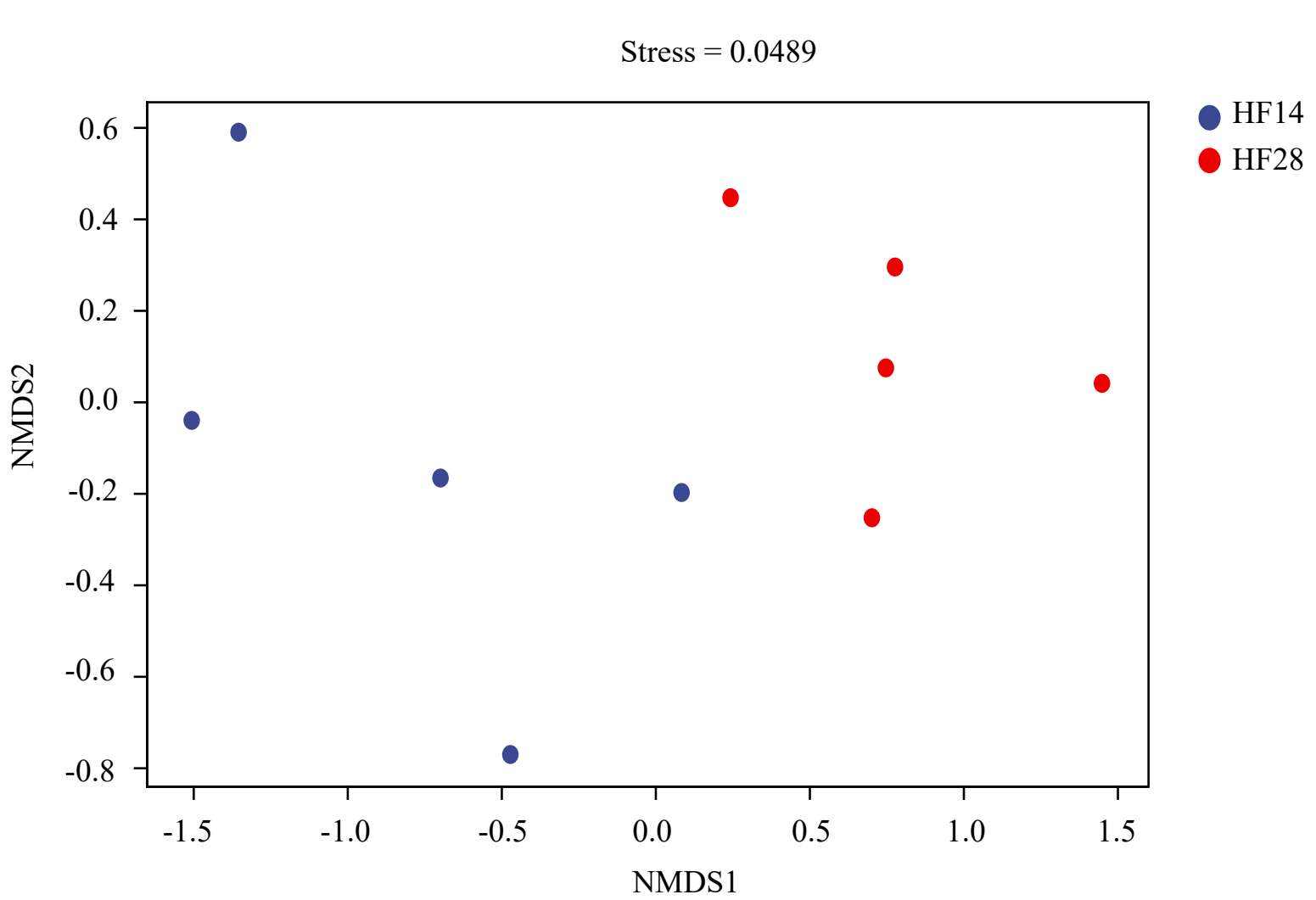

E

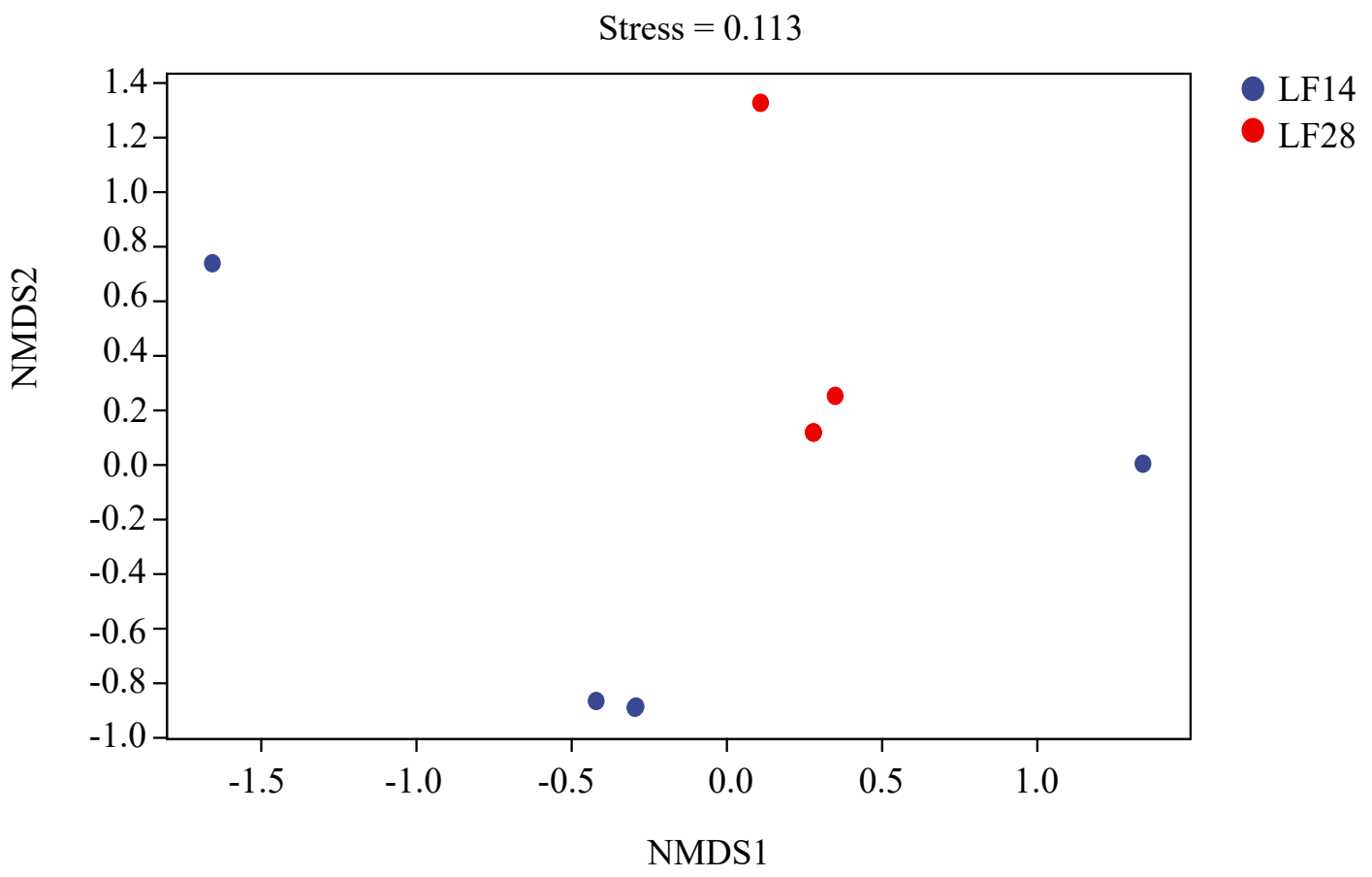

F

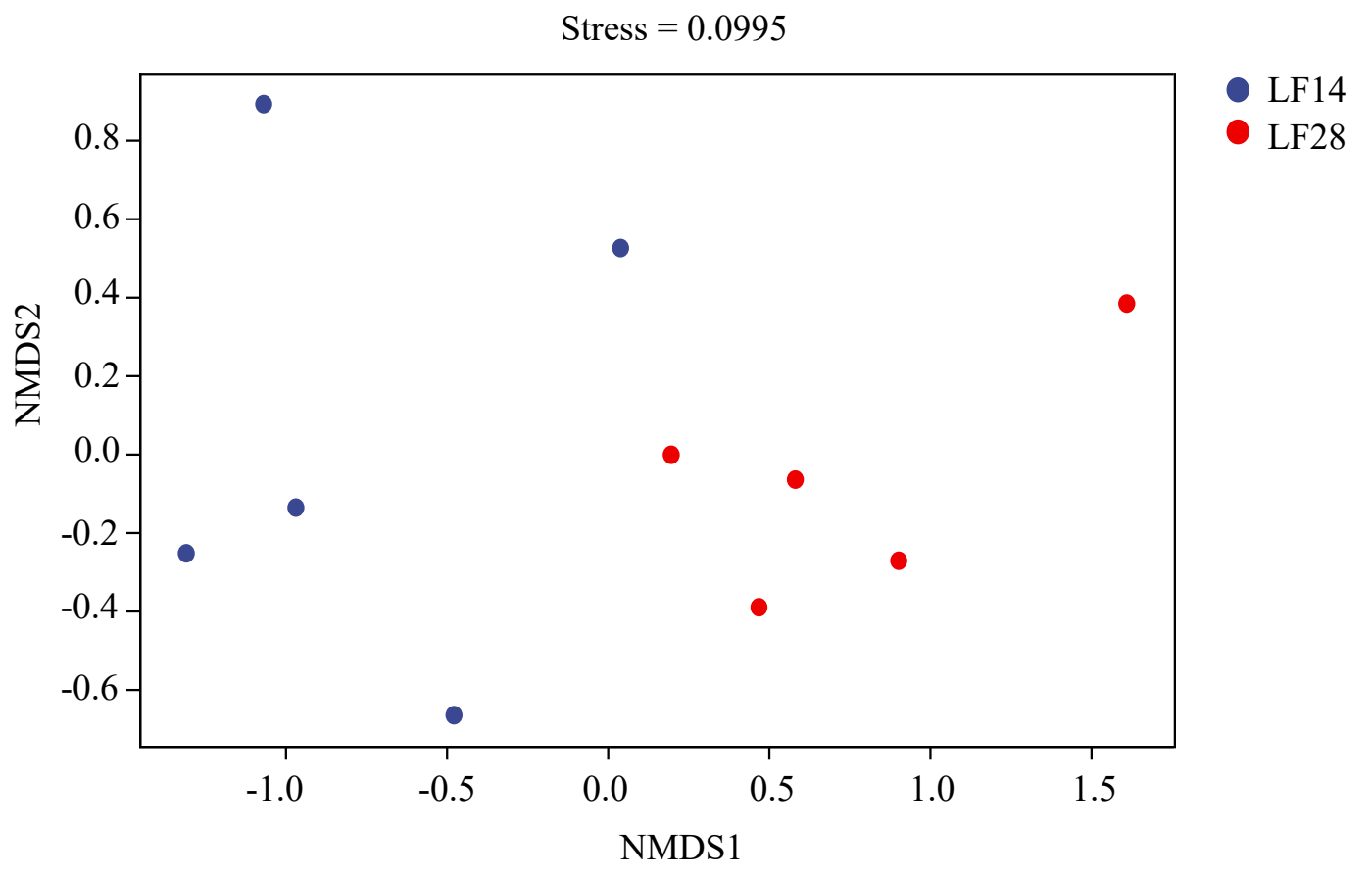

G

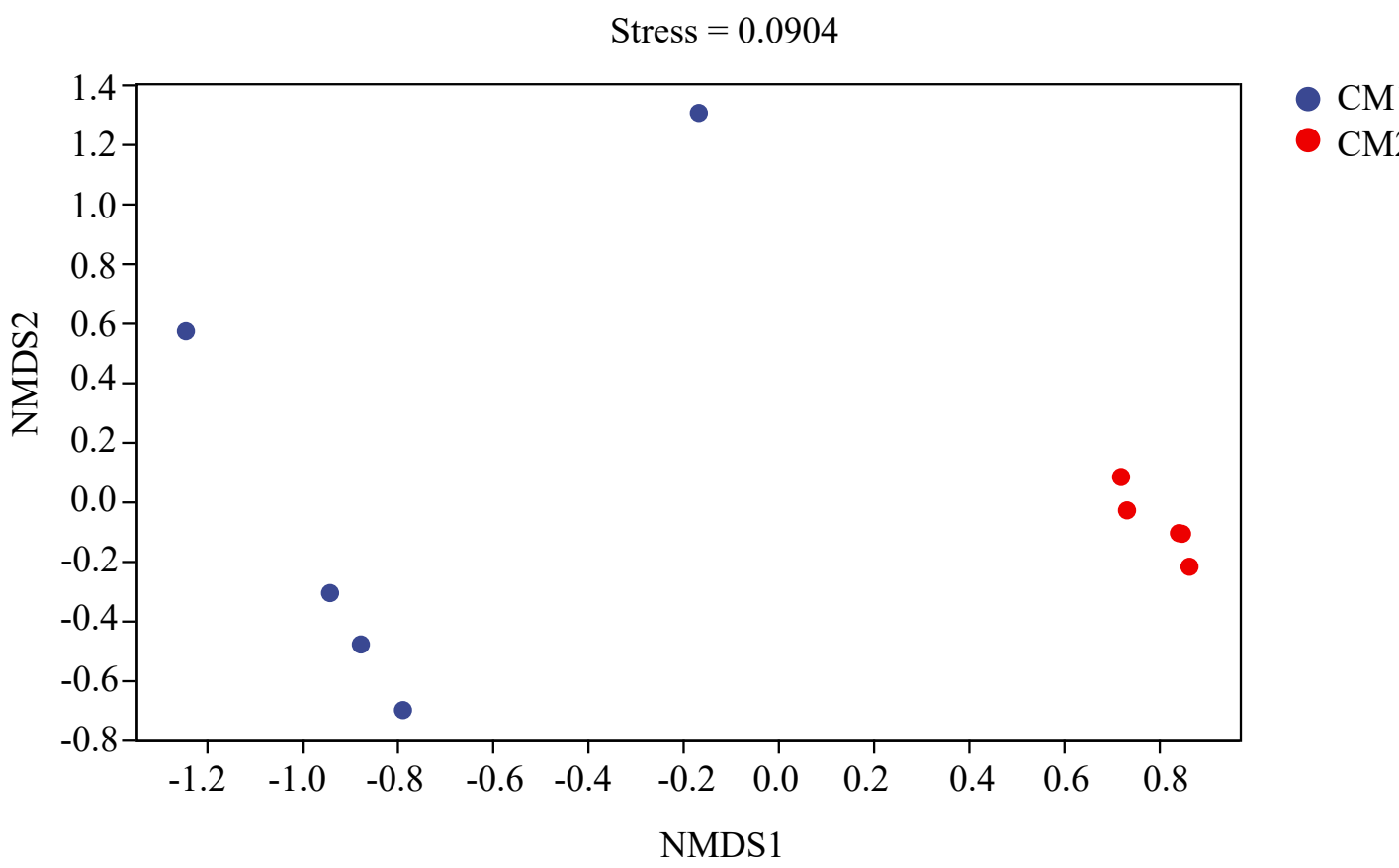

H

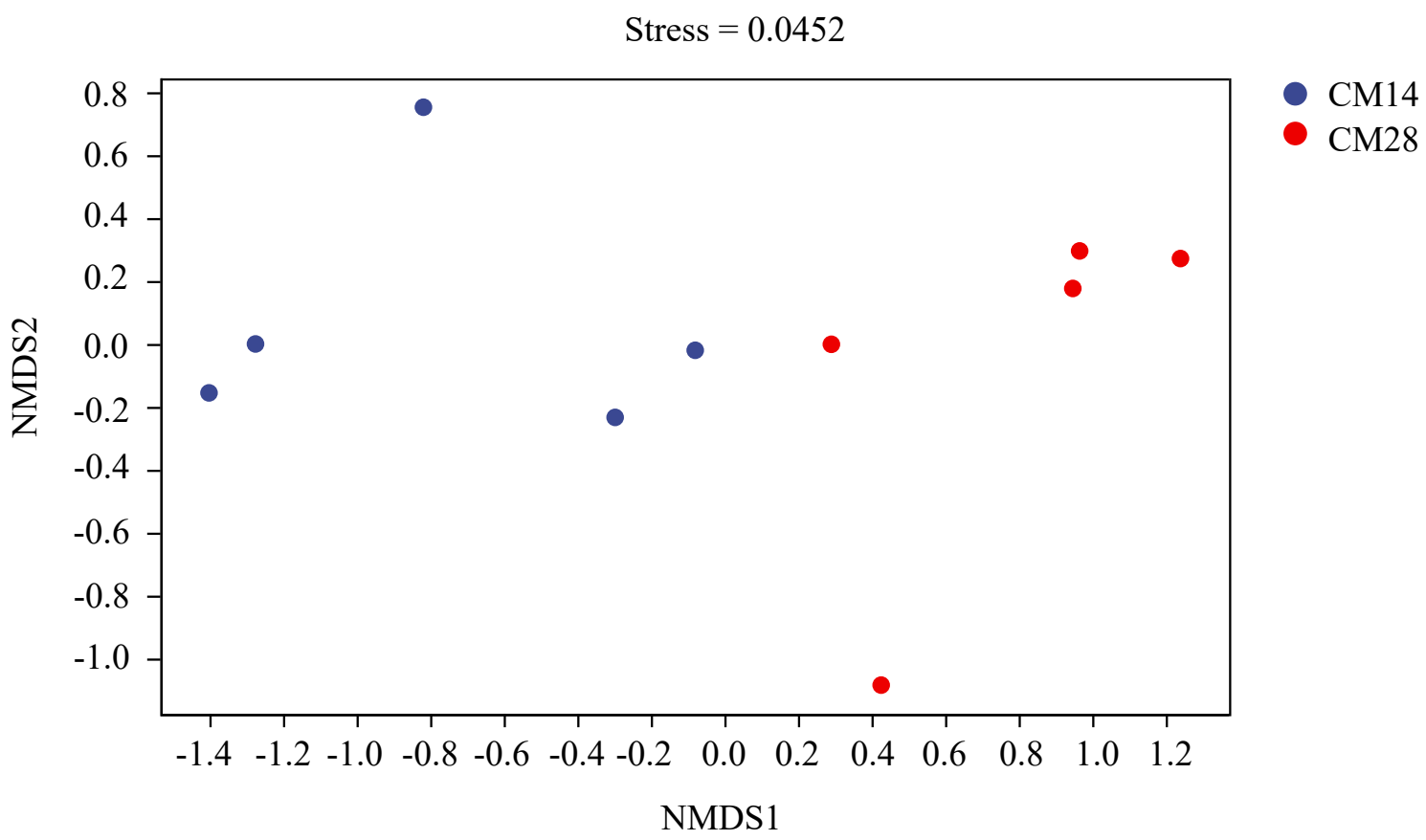

I

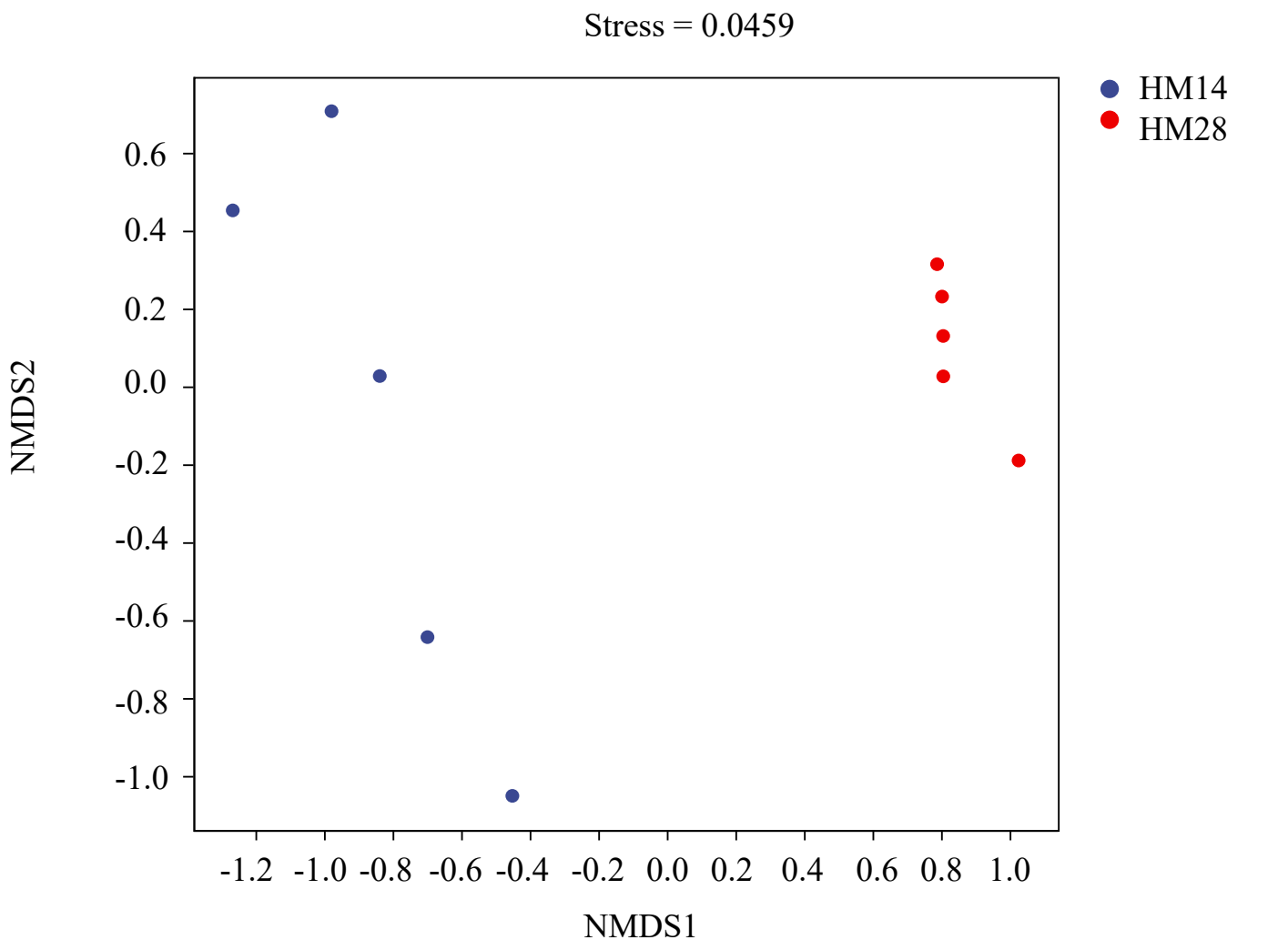

J

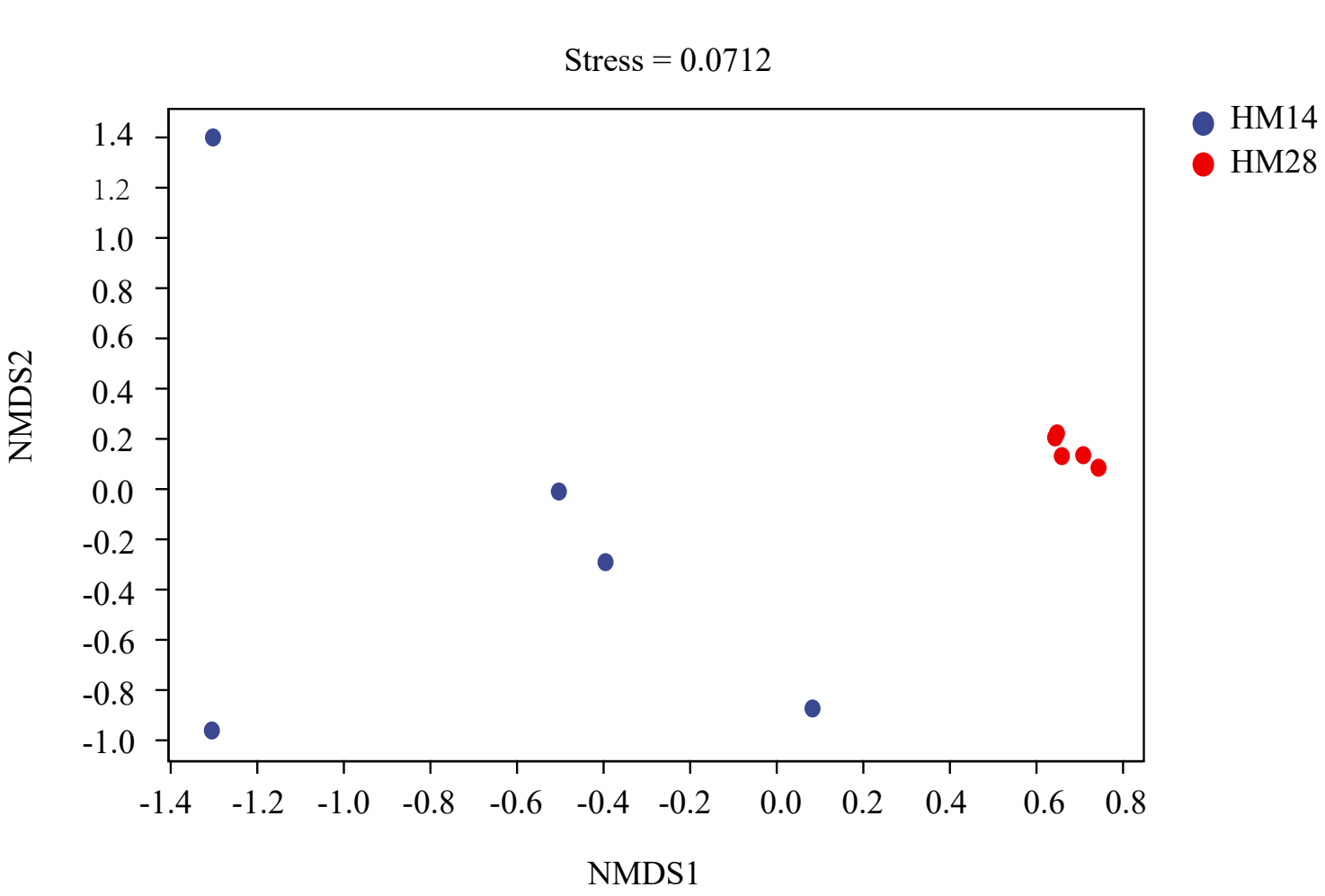

K

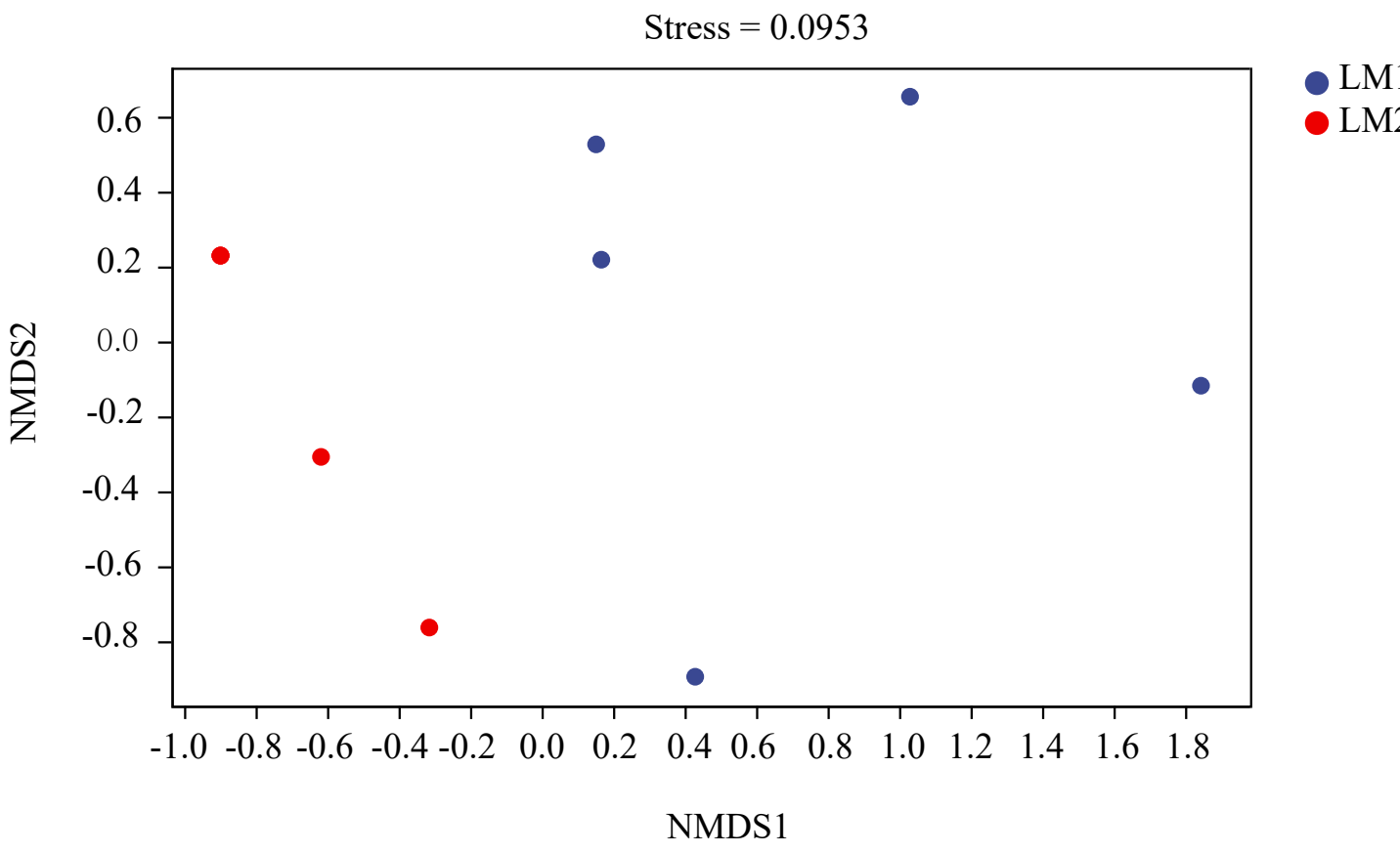

L

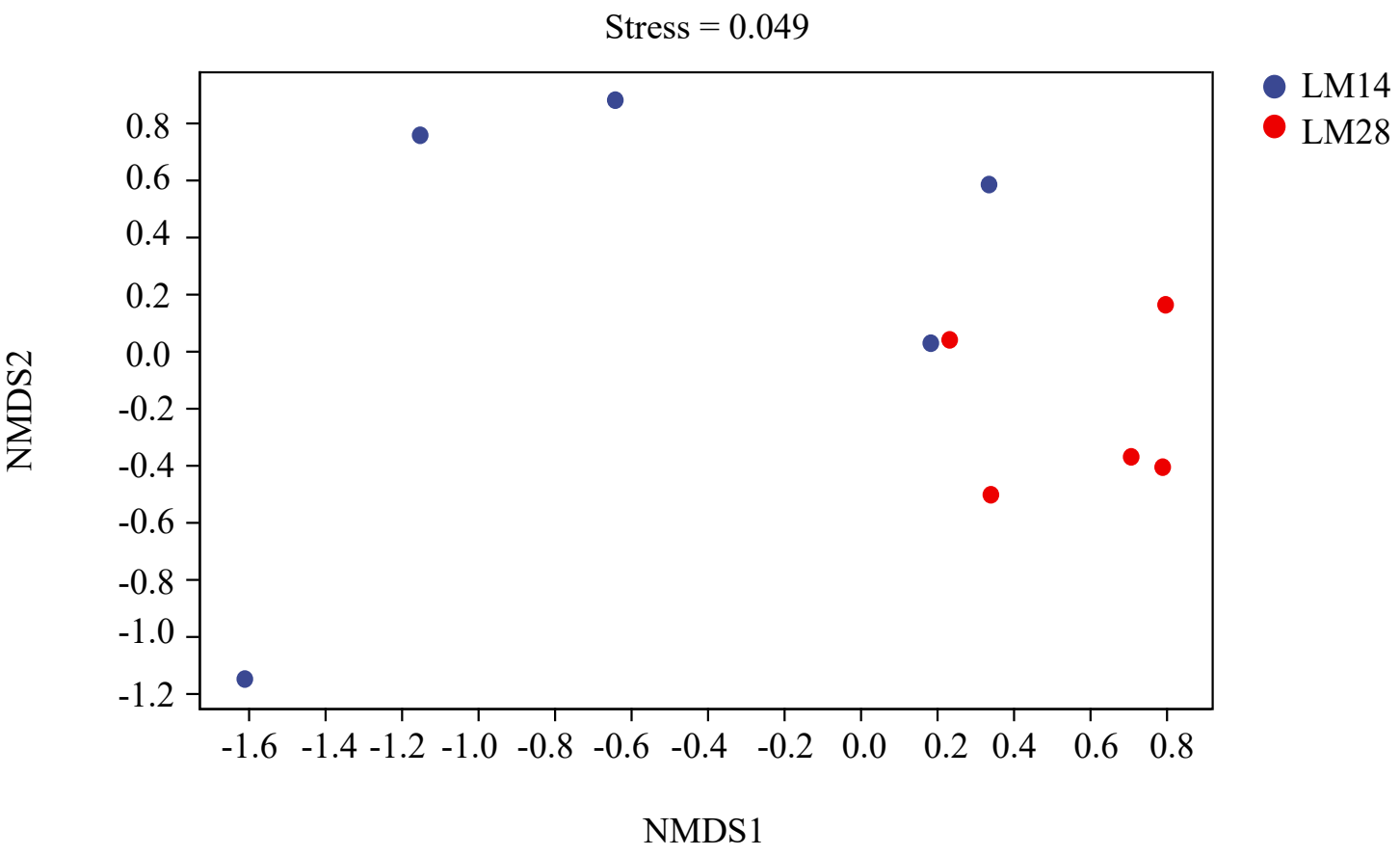

Supplement: Supplementary Figure 2 — Non-metric multidimensional scaling (NMDS) analysis based on (A,C,E) unweighted-UniFrac distances and (B,D,F) weighted-UniFrac distances plots of female Pomacea canaliculata intestine microbial communities at the different stress time in control group, high and low temperature groups, respectively. NMDS analysis based on (G,I,K) unweighted-UniFrac distances and (H,J,L) weighted-UniFrac distances plots of male P. canaliculata intestine microbial communities at the different stress time in control group, high and low temperature groups, respectively. [file Image_2.pdf]

A

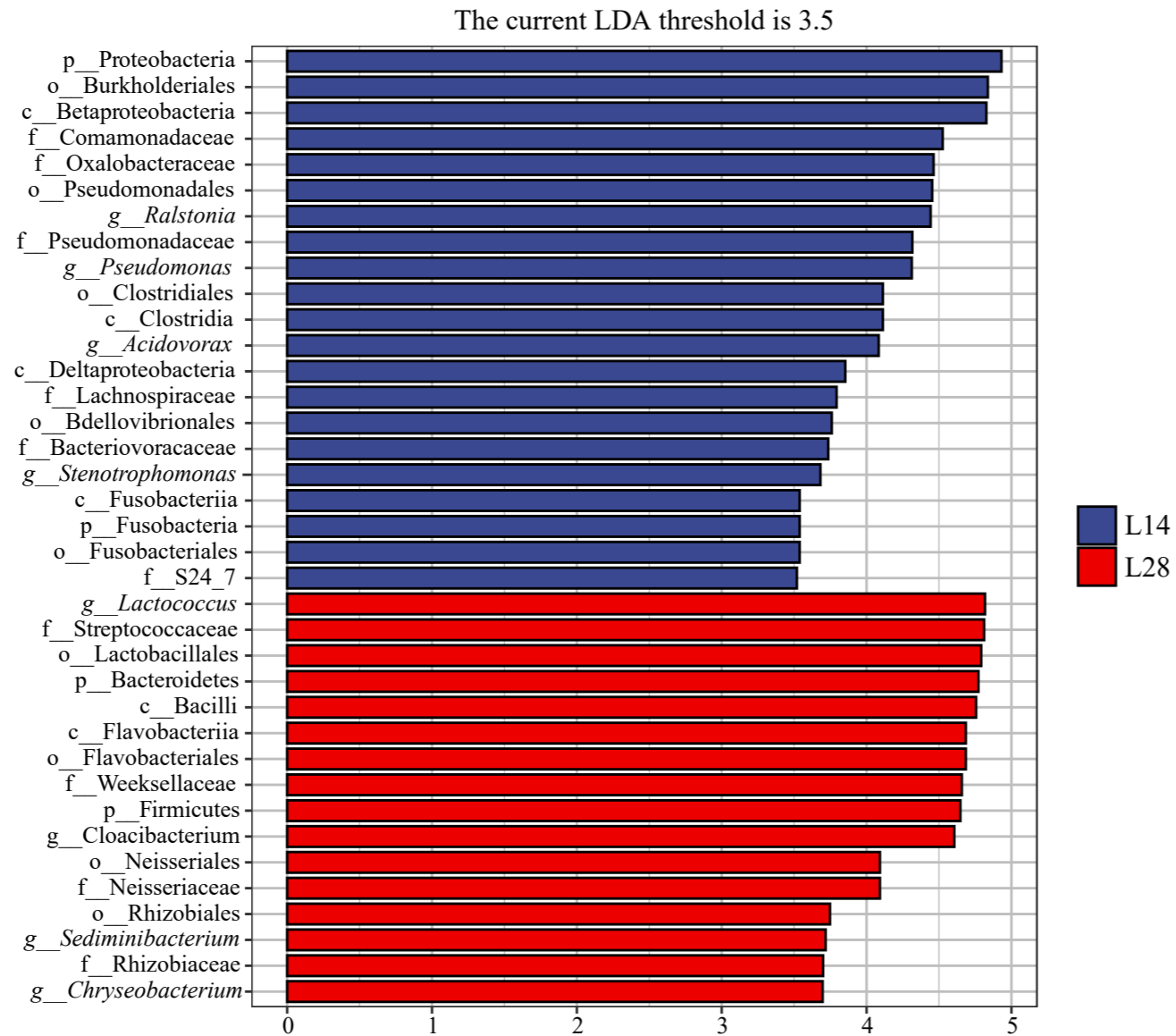

B

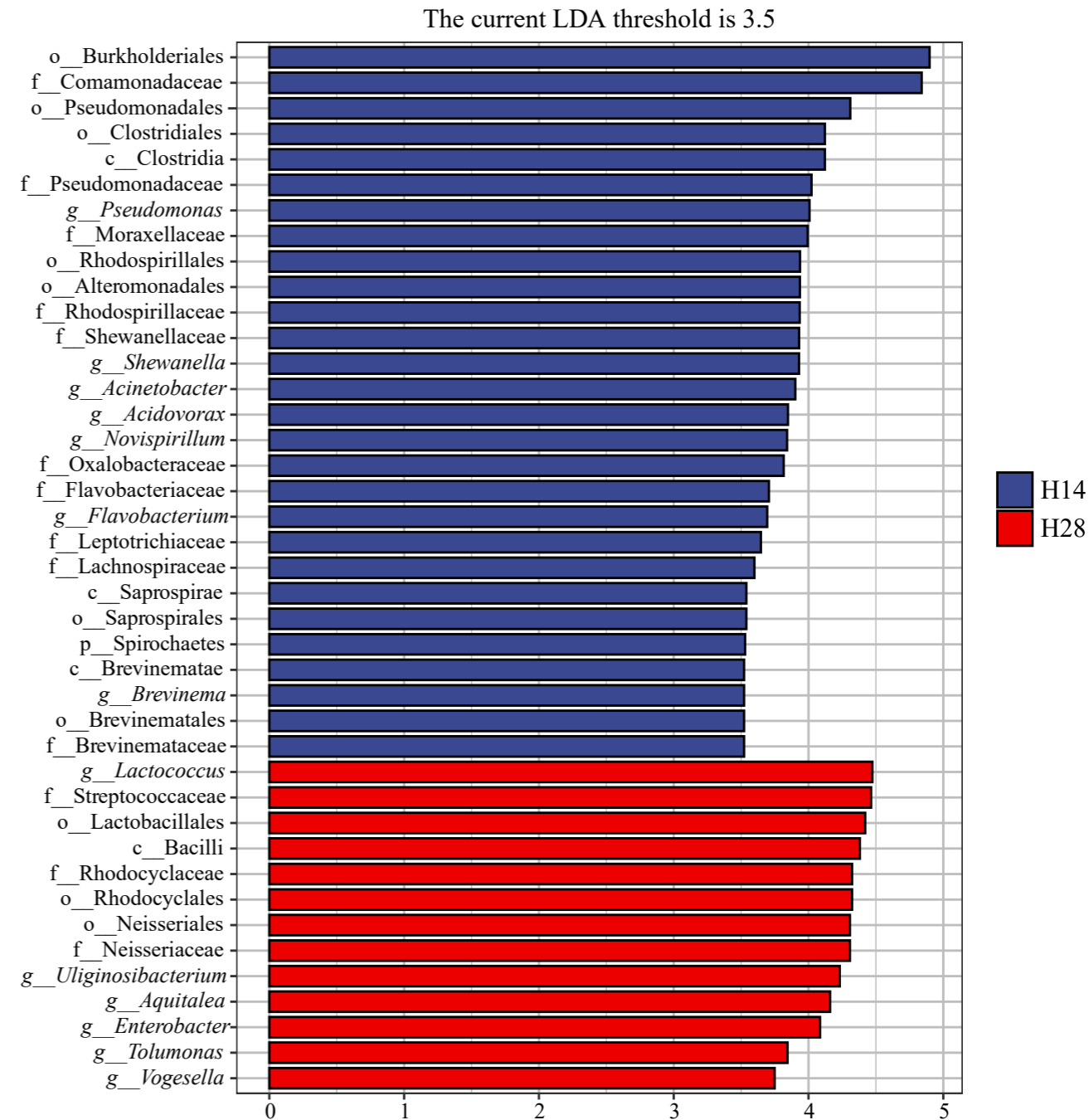

Supplement: Supplementary Figure 3 — LEfSe analysis of Pomacea canaliculata intestinal microbiota composition at different experimental time points under low (A) and high temperature (B) stress. [file Image_3.pdf]

A

The current LDA threshold is 3.5

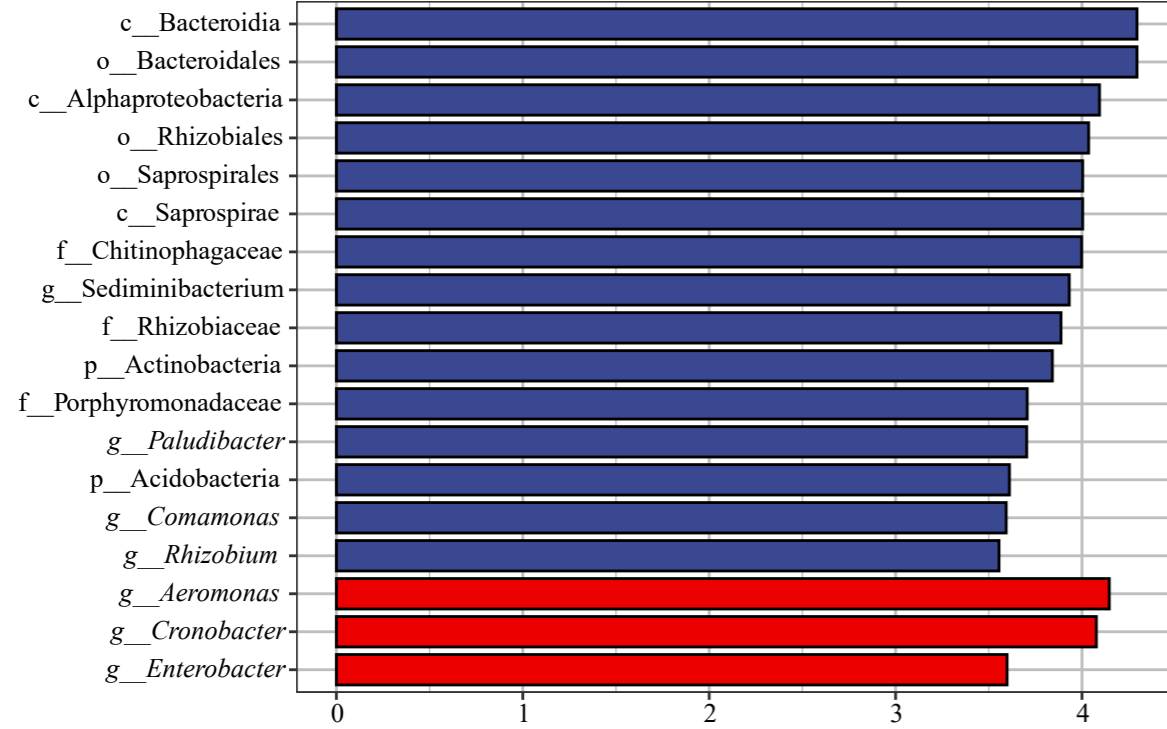

C

The current LDA threshold is 3.5

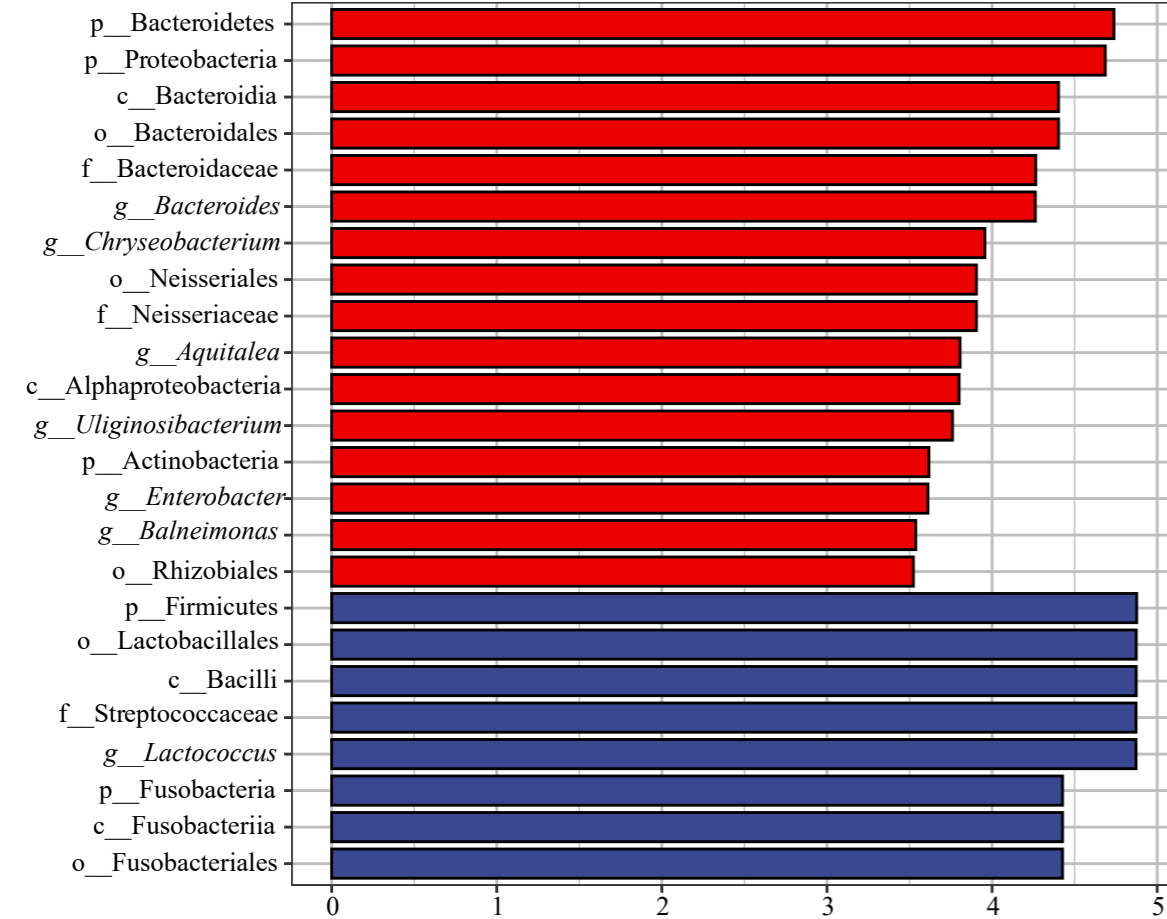

B

The current LDA threshold is 3.5

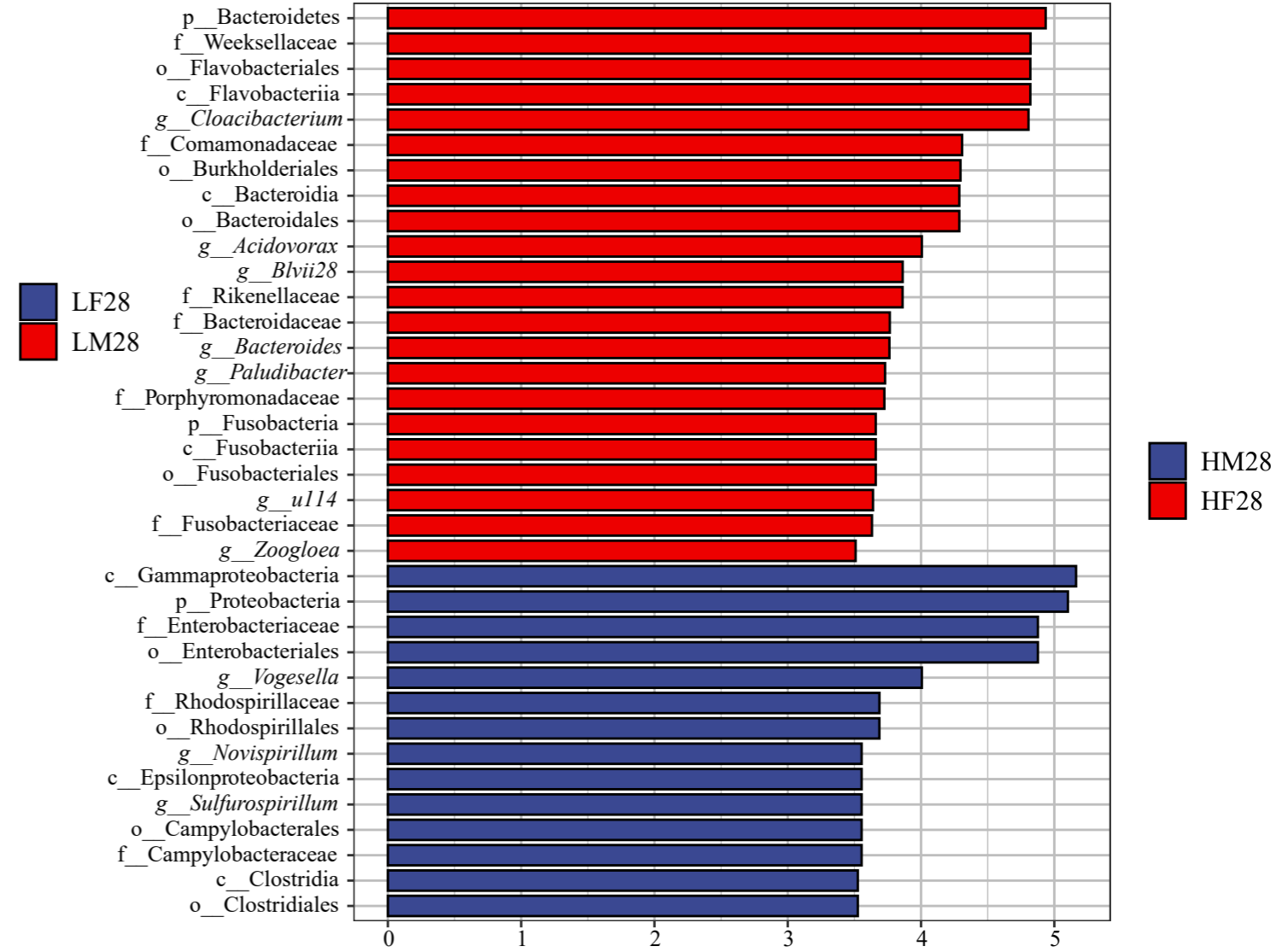

CM28  
CF28

Supplement: Supplementary Figure 4 — LEfSe analysis of female and male Pomacea canaliculata intestine microbiome under (A) high temperature, (B) low temperature, and (C) control. [file Image_4.pdf]
