# Supplementary material for: Effect of long-term temperature stress on the intestinal microbiome of an invasive snail
Source: Front Microbiol. 2022 Aug 29;13:961502. doi: 10.3389/fmicb.2022.961502 (PMC9465035; doi:10.3389/fmicb.2022.961502)
Supplement: Supplementary Table 1 — Survival rate of female and male Pomacea canaliculata at different temperature and treated time. [file Data_Sheet_1.docx]

**Table S1 |** Survival rate of female and male *Pomacea canaliculata* at different temperature and treated time.

| sex | groups | Survival rate | | | |
| --- | --- | --- | --- | --- | --- |
|  |  | 7d | 14d | 21d | 28d |
| Female | Control group (25°C) | 100% | 97.82% | 93.47% | 93.47% |
|  | Low-temperature group (15°C) | 93.48% | 93.48% | 91.30% | 91.30% |
|  | High-temperature group (35°C) | 93.48% | 82.61% | 54.35% | 45.65% |
| Male | Control group (25°C) | 100% | 100% | 97.82% | 97.82% |
|  | Low-temperature group (15°C) | 95.65% | 95.65% | 86.96% | 86.96% |
|  | High-temperature group (35°C) | 97.82% | 76.09% | 63.04% | 63.04% |

**Table S2 |** Mantel tests for the correlations between *Pomacea canaliculata* intestine microbiota and the different temperature groups using Spearman’s coefficients

| Groups | r | *P* value |
| --- | --- | --- |
| C&H | 0.378 | 0.021 |
| C&L | -0.021 | 0.492 |
| H&L | 0.159 | 0.154 |
| C14&H14 | 0.505 | 0.008 |
| C14&L14 | 0.033 | 0.348 |
| H14&L14 | 0.444 | 0.017 |
| C28&H28 | 0.516 | 0.004 |
| C28&L28 | 0.174 | 0.134 |
| H28&L28 | 0.461 | 0.012 |

**Table S3 |** Mantel tests for the correlations between *Pomacea canaliculata* intestine microbiota and the different treatment time points using Spearman’s coefficients

| Groups | r | *P* value |
| --- | --- | --- |
| C14&C28 | 0.366 | 0.024 |
| H14&H28 | 0.360 | 0.026 |
| L14&L28 | 0.021 | 0.370 |

**Table S4 |** Mantel tests for the correlations between female, male *Pomacea canaliculata* intestine microbiota and the different temperature groups using Spearman’s coefficients.

| Groups | r | *P* value |
| --- | --- | --- |
| CF&HF | 0.700 | 0.004 |
| CF&LF | 0.530 | 0.013 |
| HF&LF | 0.731 | 0.002 |
| CM&HM | 0.624 | 0.005 |
| CM&LM | 0.397 | 0.017 |
| HM&LM | 0.586 | 0.001 |
